# Robust and Efficient Authentication and Group–Proof Scheme Using Physical Unclonable Functions for Wearable Computing

**DOI:** 10.3390/s23125747

**Published:** 2023-06-20

**Authors:** Sungjin Yu, Youngho Park

**Affiliations:** 1 Electronics and Telecommunications Research Institute, Daejeon 34129, Republic of Korea; sj.yu@etri.re.kr; 2 School of Electronics and Electrical Engineering, Kyungpook National University, Daegu 41566, Republic of Korea

**Keywords:** physical unclonable function (PUF), privacy-preserving, authentication, group proof, wearable computing

## Abstract

Wearable computing has garnered a lot of attention due to its various advantages, including automatic recognition and categorization of human actions from sensor data. However, wearable computing environments can be fragile to cyber security attacks since adversaries attempt to block, delete, or intercept the exchanged information via insecure communication channels. In addition to cyber security attacks, wearable sensor devices cannot resist physical threats since they are batched in unattended circumstances. Furthermore, existing schemes are not suited for resource-constrained wearable sensor devices with regard to communication and computational costs and are inefficient regarding the verification of multiple sensor devices simultaneously. Thus, we designed an efficient and robust authentication and group–proof scheme using physical unclonable functions (PUFs) for wearable computing, denoted as AGPS-PUFs, to provide high-security and cost-effective efficiency compared to the previous schemes. We evaluated the security of the AGPS-PUF using a formal security analysis, including the ROR Oracle model and AVISPA. We carried out the testbed experiments using MIRACL on Raspberry PI4 and then presented a comparative analysis of the performance between the AGPS-PUF scheme and the previous schemes. Consequently, the AGPS-PUF offers superior security and efficiency than existing schemes and can be applied to practical wearable computing environments.

## 1. Introduction

With the development of “mobile and 5G communication” technologies, wearable computing is emerging as a new ubiquitous technology within the Internet of Things (IoT) and it has garnered a lot of attention from both scientific and academic communities [1,2,3]. The wearable devices are integrated into various types of accessories and clothing and provide useful application services in various fields, including “military, healthcare, and industry”. In particular, sustainable wearable computing technology offers innovative healthcare opportunities, which give new methods to medical professionals to treat patients. For instance, wearable computing-based healthcare systems reduce healthcare costs and provide various medical services, including “monitoring, medical consultation, and emergency treatment” [4].

In these environments, wearable devices collect medical data, including “asthma level, blood pressure, electrocardiogram, body temperature” from the patients, and then transmit the corresponding data to the paired mobile terminal. The mobile terminal transmits the received data to the trusted cloud server, and authorized medical professionals remotely connect to the trusted cloud server and precisely monitor, analyze, and diagnose the health data of patients stored within the server. However, despite the numerous advantages of wearable computing, there are several difficulties and challenges that need to be addressed [5]. In wearable computing environments, serious security and privacy issues may arise since the messages are transmitted via an insecure channel [6]. If the collected data from the wearable devices are exposed, an adversary can obtain the sensitive information of legitimate patients and may attempt potential cyber security attacks. Hence, adversaries can bring many unexpected threats and jeopardize the patients’ lives by transmitting false medical diagnoses, such as “treatments and medications”. In addition to cyber security attacks, wearable devices cannot prevent physical threats since they are deployed in hostile and unattended circumstances. Furthermore, considering the resource limitations of wearable devices, it is suitable to adopt lightweight cryptographic primitives, such as “hash functions and symmetric key cryptography that require low computation and communication costs [7]”. In wearable computing environments, it is essential to identify whether data collected from multiple wearable devices belong to the same authorized user. Thus, a lightweight privacy-preserving authentication and group–proof scheme is indispensable to ensure simultaneous identification and secure communication in wearable computing environments.

Recently, Guo et al. [8] presented an “anonymous authenticated key agreement and group–proof protocol for wearable computing” to provide secure communication and simultaneous identification. Guo et al. claimed that their scheme was protected against physical/cyber security attacks, including “physical wearable device capture, impersonation, and forgery” attacks, and guaranteed “secure mutual authentication and untraceability”. Unfortunately, we prove that Guo et al.’s scheme was not protected against security attacks, such as “session key disclosure, man-in-the-middle (MITM), and impersonation” attacks, and it does not offer several security properties, including “untraceability and mutual authentication”. Hence, we present a “new efficient and robust authentication and group–proof scheme using physical unclonable functions (PUF) for wearable computing”, denoted as AGPS-PUFs, to address the security issues of Guo et al.’s scheme [8].

### 1.1. Motivations

The main purpose of this paper is to identify and improve the security problems of Guo et al.’s scheme based on the threat model presented by them. This paper proves that their protocol [8] is not protected against lethal security attacks and does not offer sensitive security features in wearable computing environments. Guo et al. [8] designed a high-security-supported cryptographic and efficient group–proof scheme for wearable computing. However, they should have examined their protocol from the point of view that we analyzed and proved. This fact motivated us to design a “new efficient and robust authentication and group–proof scheme using PUF for wearable computing”. This scheme is resilient to lethal security attacks and drawbacks that exist in wearable computing environments while guaranteeing security functionalities.

### 1.2. Research Contributions

This section introduces the main contribution of the AGPS-PUF.

- The AGPS-PUF is specifically designed to improve the security vulnerabilities of Guo et al.’s scheme and offers reliable authentication and maintenance for wearable computing. The AGPS-PUF carries out mutual authentication between a mobile user and wearable devices through a trusted entity known as the cloud server. The PUF enables wearable devices to resist tampering, including physical security attacks.
- We propose the protocol and demonstrate its effectiveness and security strengths via informal and formal security analyses. We exploited the well-known “AVISPA simulation” [9] and “ROR Oracle model” [10].
- We prove that the AGPS-PUF offers efficient performance in terms of security functionalities and overheads, as compared to previous schemes explored in the literature.

### 1.3. Paper Outlines

The rest of the paper is organized as follows. Section 2 presents the related works for wearable computing environments. Section 3 introduces the preliminaries. In Section 4 and Section 5, we review Guo et al.’s scheme [8] and then prove the security shortcomings of Guo et al.’s scheme. Section 6 designs a “new PUF-based privacy-preserving authentication and group–proof scheme for wearable computing” to resolve the security problems of Guo et al.’s scheme. Section 7 analyzes the security of the AGPS-PUF when performing formal and informal security analyses. Section 8 introduces the testbed experiments for cryptographic operations using MIRACL crypto SDK. Section 9 analyzes the performance comparison of the AGPS-PUF with related schemes. Finally, Section 10 summarizes the future works and conclusions of this paper.

## 2. Related Works

Over the last few years, many authentication and key agreement (AKA) schemes have been presented for wearable computing to ensure privacy for legitimate users [11,12,13]. The public key cryptosystem (PKC)-based AKA schemes consist of three mechanisms: “traditional PKC scheme [14], identity-based PKC scheme [15], and certificateless PKC scheme [16]”. The traditional PKC scheme faces problems in managing user certificates and needs high computing capabilities, so it is not applicable to wearable computing environments with constrained resources. Identity-based PKC schemes deal with the difficulty of certificate management; however, they are presented for server–client environments. The certificateless PKC scheme enhances the key escrow problem of the identity-based PKC scheme and prevents certificate management and delivery problems from the traditional PKC scheme [17]. However, these existing PKC-based AKA schemes [14,15,16] are not suitable for wearable computing environments because they utilize PKC, such as elliptic curve cryptography (ECC) and bilinear pairing, which require high communication and computation overheads.

The design of a lightweight AKA scheme for wearable computing environments has garnered a lot of attention due to the efficiency problem of the PKC-based AKA scheme and constrained resources for IoT and sensor devices. The lightweight AKA scheme has two main features: “password-based two-factor AKA scheme or password and biometric-based three-factor AKA scheme”. These AKA schemes utilize lightweight cryptographic primitives, including the “one-way hash function, XOR operation, and symmetric key cryptography”. Recently, numerous lightweight AKA schemes [18,19,20] were designed for wearable computing environments to provide useful services with lightweight properties. Li et al. [21] proposed a “secure AKA scheme with user anonymity and lightweight for healthcare applications” in wireless medical sensor networks (WMSN). Unfortunately, Das et al. [22] demonstrated that Li et al.’s scheme [21] is insecure to “privileged insider and sensor node capture” attacks and fails to ensure “user anonymity”. Wu et al. [23] presented an “enhanced two-factor assisted AKA scheme in WMSN environments”. Wu et al. [23] claimed that their protocol is resilient to lethal security attacks and offers the necessary security features. Unfortunately, Srinivas et al. [24] proved that their scheme [23] is not resistant to lethal security attacks, such as “stolen smart card, offline password guessing, user impersonation, and denial of service (DoS)” attacks. Srinivas et al. [24] proposed an “efficient and reliable AKA scheme for healthcare services with WMSN” to address the security weaknesses of Wu et al.’s scheme [23]. Amin et al. [25] designed a “lightweight and anonymous two-factor based AKA scheme” to provide secure patient data in patient monitoring systems for WMSN. Unfortunately, Ali et al. [26] analyzed Amin et al.’s scheme [25] and found that it does not prevent “known-session key temporary information, user impersonation, and offline password guessing” attacks. Ali et al. [26] presented an “enhanced biometric-based three-factor AKA scheme for healthcare monitoring in WMSN” to resolve the security shortcomings of Amin et al.’s scheme [25]. Gupta et al. [27] designed a “lightweight AKA scheme for wearable devices with user anonymity”. Gupta et al.’s scheme [27] has high scalability because the wearable sensing device registration phase does not need a secure channel. However, Hajian et al. [28] proved that Gupta et al.’s scheme [27] is not resistant to lethal security attacks, including “compromise sensing device, desynchronization, and privileged insider” attacks. Hajian et al. [28] proposed a “scalable and lightweight three-factor based AKA scheme with user-friendly and anonymous for wearable sensing devices” to improve the security problems of Gupta et al.’s scheme [27]. However, Yu et al. [29] pointed out that their protocol [28] is still not resistant to “mobile device stolen”, “session key disclosure, MITM, impersonation” attacks and does not guarantee “mutual authentication”. Unfortunately, these lightweight AKA schemes for wearable computing do not identify whether the collected data from multiple wearable devices belong to the same authorized user.

Guo et al. [8] designed an “anonymous and lightweight AKA and group–proof scheme for wearable computing”, which can verify that multiple wearable devices belong to the same user. Guo et al. [8] claimed that their protocol ensures secure data transmission between each entity and is resilient to lethal security attacks. However, based on the threat model presented by them, we have proven that Guo et al.’s scheme [8] is vulnerable to lethal security threats, such as “impersonation, MITM, and session key disclosure” attacks, and does not offer several security properties, such as “untraceability and mutual authentication”. In addition to cyber security attacks, wearable devices may be fragile to physical threats since they are batched in insecure circumstances. Therefore, we propose an “efficient and robust authentication and group-proof scheme using the PUF for wearable computing” to supplement the security functionalities and address the security shortcomings of Guo et al.’s scheme [8].

## 3. Preliminaries

The following provides an overview of the preliminaries.

### 3.1. Threat Model

We introduce the adversary capabilities based on the “Dolev-Yao (DY) model” [30,31].

- An adversary (henceforth denoted as A) can “resend, eavesdrop, block, and delete” the exchanged messages over an insecure channel.
- A can steal the mobile device (MD) and the wearable device (WD) of the legitimate user. However, A cannot simultaneously capture the MD and WD of the legitimate user. The cloud server and registration center are trusted authorities and cannot be compromised by A.
- A can extract the secret information stored in the captured MD or WD by performing the “power-analysis attacks” [32] and “physical capture attacks” [33].

### 3.2. PUF

The PUF [34,35] is a physical circuit that manufactures an output of a physical microstructure. The PUF does not store a private key in the smart device and it is extremely difficult to clone the circuit. The PUF utilizes an input/output bit string pair, denoted as the challenge/response pair. Even if various challenges occur in the PUF circuit, each has a unique output response. The PUF preserves smart devices in IoMT-enabled TMIS environments from side-channel and tampering threats. The PUF is expressed through a process denoted as R=PUF(C), where *C* and *R* are the challenge/response. The following are several properties of the PUF.

- The PUF is easy to implement and evaluate.
- The PUF relies on the system’s physical microstructure.
- Any attempt to tamper with a smart device that contains the PUF will update the behavior of the PUF and, thus, destroy it [36].

**Figure 1:**
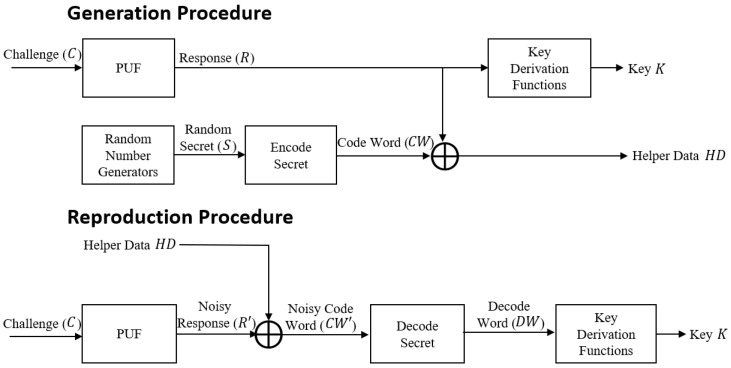
Key generator mechanism of the PUF.

Figure 1 shows a “PUF-based key generator procedure”. As shown in Figure 1, the PUF generates strong extractors for a private secret key based on various functions, including “encode, decode, and key derivation” functions. Thus, the PUF makes it impossible for attackers to perform lethal physical threats. Moreover, these properties combine to make a “good solution for the robust and efficient authentication of lightweight devices in wearable computing environments”.

### 3.3. System Model

**Figure 2:**
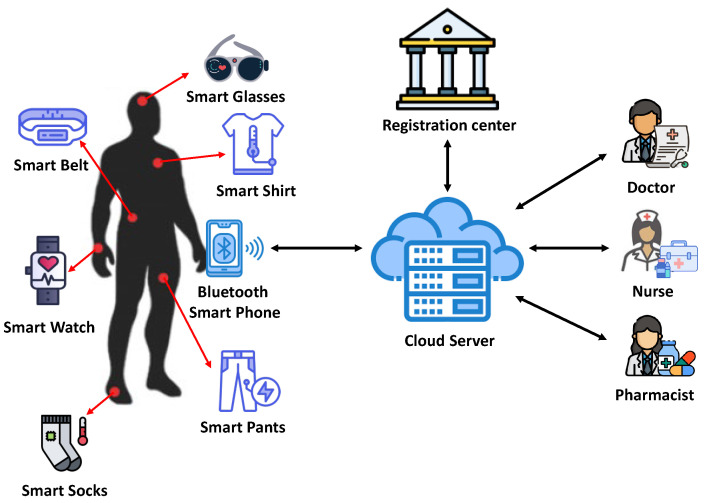
System model for wearable computing.

This section introduces an overview of the system model (see Figure 2) of this paper. The system model for wearable computing is composed of four entities: registration center, cloud server, mobile users, and wearable devices.

- Registration center: This entity is a trusted authority that registers wearable devices and mobile users in a secure channel. Moreover, the registration center sets the secret credentials of each wearable device before being batched in wearable computing environments.
- Cloud server: This entity is also a trusted authority. The cloud server stores and shares the health data of legitimate patients and has computational and storage capabilities to manage patients’ health data.
- Mobile users: They have a mobile terminal and wear wearable devices to analyze the health status of the patients. The mobile terminal receives health data from the wearable devices, and then sends the received data to the cloud server through wireless communications. Moreover, remote authorized users access the cloud server to analyze the patients’ data and provide accurate medical diagnoses based on the stored physiological data.
- Wearable device: Wearable devices track and collect health data from corresponding body parts of patients. Then, the collected data are transmitted to the paired mobile terminal via Bluetooth.

## 4. Review of Guo et al.’s Scheme

This section introduces the reviews of Guo et al.’s scheme [8]. Table 1 shows the notations utilized in this article.

### 4.1. System Setup Phase

In this section, RC denotes the secret credentials of each $WD_{j}$.

**SP-1:** 
RC select s a master private key MK for CS.
**SP-2:** 
RC chooses a unique identity $ID_{WD}$ for each $WD_{j}$ and computes the pseudo-identity $PID_{WD}=h(ID_{WD}\left||MK\right)$. After that, RC generates a temporary identity $TID_{WD}$ for each $WD_{j}$.
**SP-3:** 
RC stores $\left\{ID_{WD},TID_{j}^{old}=null,TID_{j}^{new}=TID_{WD}\right\}$ in CS’s secure database and then stores $\left\{PID_{WD},TID_{WD},h\left(\cdot \right)\right\}$ in the memory of $WD_{j}$.

### 4.2. User Registration Phase

In this phase, $U_{i}$ registers with RC and obtains certain secret information to utilize later for authentication.

**URP-1:** 
$U_{i}$ selects $ID_{U}$ and $PW_{i}$ at $MD_{i}$ and $WD_{j}$. After that, $MD_{i}$ generates a random number $RN_{i}$ and computes $HID_{U}=h(ID_{U}||RN_{i})$ and $HPW_{i}=h(PW_{i}||RN_{i})$. Then, $MD_{i}$ sends $\left\{HID_{U},HPW_{i}\right\}$ to RC over a secure channel.
**URP-2:** 
RC selects a temporary identity $TID_{U}$ and a random number $r_{i}$. After that, RC calculates $Auth^{*}=h(HID_{U}||HPW_{i}\left|\right|r_{i})$, $A_{i}=r_{i}⊕h\left(HID_{U}⊕HPW_{i}\right)$, $B_{i}=h(TID_{U}\left|\right|r_{i}\left||MK\right)$, $C_{i}=r_{i}⊕h(TID_{U}\left||MK\right)$. Finally, RC stores $\left\{TID_{j}^{old}=null,TID_{j}^{new}=TID_{WD},C_{i}\right\}$ in CS’s secure database and then sends $\left\{Auth^{*},TID_{U},A_{i},B_{i}\right\}$ to $MD_{i}$ over a secure channel.
**URP-3:** 
$MD_{i}$ computes $HAuth=h(Auth^{*}||HPW_{i}⊕RN_{i})$, $D_{i}=RN_{i}⊕A_{i}$, $E_{i}=B_{i}⊕h\left(TID_{U}⊕HPW_{i}⊕RN_{i}\right)$, and $F_{i}=RN_{i}⊕h(ID_{U}||PW_{i})$. Finally, $MD_{i}$ stores $\left\{TID_{U},HAuth,D_{i},E_{i},F_{i},h\left(\cdot \right)\right\}$ in its memory.

### 4.3. Login and Authentication Phase

In this phase, all participants authenticate each other and establish a common session key.

**LAP-1:** 
$U_{i}$ first inputs $ID_{U}$ and $PW_{i}$ into $MD_{i}$. After that, $MD_{i}$ computes $RN_{i}=F_{i}⊕h(ID_{U}||PW_{i})$, $HID_{i}^{*}=h(ID_{U}||RN_{i})$, $HPW_{i}^{*}=h(PW_{i}||RN_{i})$, $R_{i}^{*}=A_{i}⊕h\left(HID_{i}^{*}⊕HPW_{i}^{*}\right)=D_{i}⊕RN_{i}⊕h\left(HID_{i}^{*}⊕HPW_{i}^{*}\right)$, $Auth^{*}=h(HID_{i}^{*}||HPW_{i}^{*}\left|\right|R_{i}^{*})$, and $HAuth^{*}=h\left(Auth^{*}⊕HPW_{i}^{*}⊕RN_{i}\right)$, and checks $HAuth^{*}\overset{?}{=}HAuth$. If it matches, $MD_{i}$ generates a random nonce $n_{1}$ and a current timestamp $T_{1}$ and then transmits $Msg_{1}=\left\{n_{1},T_{1}\right\}$ to $WD_{j}$ over an insecure channel.
**LAP-2:** 
$WD_{j}$ verifies the freshness of $|T_{2}-T_{1}|\;\leq \;\Delta T_{i}$, where $T_{2}$ is the current timestamp and $\Delta T_{1}$ is the maximum transmission delay for the message to be transmitted between $MD_{i}$ and $WD_{j}$. If they match, $WD_{j}$ selects $n_{2}$ and computes $M_{1}=h(TID_{WD}||PID_{WD})⊕n_{2}$ to transmit the secret parameters securely, and $M_{2}=$$h(M_{1}||T_{2}||n_{1}||n_{2})$ to verify the authorized entity. After that, $WD_{j}$ transmits $Msg_{2}=\left\{M_{1},M_{2},TID_{WD},T_{2}\right\}$ to $MD_{i}$.
**LAP-3:** 
$MD_{i}$ checks the freshness of $|T_{3}-T_{2}|\;\leq \;\Delta T_{i}$. If the condition is met, $MD_{i}$ selects $n_{3}$ and computes $B_{i}=E_{i}⊕h\left(TID_{U}⊕HPW_{i}⊕RN_{i}\right)$, $M_{3}=n_{3}⊕h(TID_{U}\left|\right|r_{i}\left|\right|T_{3})$ to transmit the random nonce securely, $M_{4}=h(TID_{U}\left|\right|B_{i}\left|\right|n_{3}\left|\right|T_{3})$ to verify the authorized entity, $M_{5}=h(TID_{U}||TID_{WD})⊕n_{1}$ to transmit the secret parameters securely, and $M_{6}=h(TID_{U}||TID_{WD}\left|\right|M_{2}\left|\right|M_{4}\left|\right|n_{3}\left|\right|T_{3})$ to verify the authorized entity, and sends $Msg_{3}=\left\{M_{1},M_{2},M_{3},M_{4},M_{5},M_{6},TID_{U},TID_{WD},T_{2},T_{3}\right\}$ to CS over an insecure channel.
**LAP-4:** 
CS verifies the freshness of $|T_{4}-T_{3}|\;\leq \;\Delta T_{i}$. If it matches, CS retrieves $TID_{U}$ in the database. There are three scenarios for $TID_{U}$. The first scenario is $TID_{i}^{old}=TID_{U}$, indicating that CS and $U_{i}$ did not correctly update the temporary identity of $U_{i}$ in the previous session. The second scenario is $TID_{i}^{new}=TID_{U}$, indicating that CS and $U_{i}$ correctly updated the temporary identity of $U_{i}$ in the previous session. In the third scenario, there is no matching of $TID_{U}$ in the CS database, and the authentication phase is terminated. For the first two scenarios, CS obtains $\left\{C_{i}\right\}$, corresponding to $TID_{U}$ in its database. After that, CS computes $r_{i}^{*}=C_{i}⊕h(TID_{U}\left||MK\right)$, $B_{i}^{*}=h(TID_{U}\left|\right|r_{i}^{*}\left||MK\right)$, $n_{3}^{*}=M_{3}⊕h(TID_{U}\left|\right|r_{i}^{*}\left|\right|T_{3})$, $M_{4}^{*}=h(TID_{U}\left|\right|B_{i}^{*}\left|\right|n_{3}^{*}\left|\right|T_{3})$, and $M_{6}^{*}=h(TID_{U}||TID_{WD}\left|\right|M_{2}\left|\right|M_{4}^{*}\left|\right|n_{3}^{*}\left|\right|T_{3})$, and verifies $M_{4}^{*}\overset{?}{=}M_{4}$ and $M_{6}^{*}\overset{?}{=}M_{6}$. If they are not equal, the authentication phase is terminated. Otherwise, CS successfully authenticates $U_{i}$ and then updates the temporary identity of $U_{i}$. For the second scenario, $U_{i}$’s new temporary identity remains unchanged for the time being and is updated later in the session.
**LAP-5:** 
CS retrieves $TID_{WD}$ in its database. Similar to LAP-4, there are three scenarios: $TID_{j}^{old}=TID_{WD}$, $TID_{j}^{new}=TID_{WD}$, or $TID_{WD}$ cannot be found in the CS database. In the first two scenarios, CS obtains $\left\{ID_{WD}\right\}$, corresponding to $TID_{WD}$ in its database, and then computes $PID_{WD}=h(ID_{WD}\left||MK\right)$, $n_{2}^{*}=h(TID_{WD}||PID_{WD})⊕M_{1}$, $n_{1}^{*}=h\left(TID_{U}⊕TID_{WD}\right)⊕M_{5}$, and $M_{2}^{*}=h(M_{1}\left|\right|T_{2}\left|\right|n_{1}^{*}\left|\right|n_{2}^{*})$, and checks $M_{2}^{*}\overset{?}{=}M_{2}$. If it matches, CS successfully authenticates $WD_{j}$. Then, CS updates the temporary identity of $WD_{j}$ as it updates $U_{i}$’s temporary identity.
**LAP-6:** 
CS selects $n_{4}$ and timestamp $T_{4}$. After that, CS computes $M_{7}=B_{i}⊕T_{4}=$$h(TID_{U}\left|\right|r_{i}\left||MK\right)⊕T_{4}$ to transmit the secret parameters securely, $M_{8}=n_{4}⊕h(TID_{U}\left|\right|r_{i}\left|\right|n_{3}\left|\right|T_{4})$ to transmit the secret parameters securely, $SK_{CS-U}=h(TID_{U}\left|\right|M_{7}\left|\right|n_{3}\left|\right|n_{4}\left|\right|T_{3}\left|\right|T_{4})$, $M_{9}=h(M_{4}\left|\right|M_{8}||SK_{CS-U}\left|\right|T_{3}\left|\right|T_{4})$ to verify the authorized entity, $SK_{WD-U}=h(TID_{U}||TID_{WD}||h(ID_{WD}\left||MK)|\right|n_{2}\left|\right|n_{4}\left|\right|T_{4})$, $M_{10}=h(M_{4}\left|\right|M_{8}||SK_{WD-U}\left|\right|T_{3}\left|\right|T_{4})$ to verify the authorized entity, and $M_{11}=SK_{WD-U}⊕h(B_{i}\left|\right|n_{1}\left|\right|n_{3}\left|\right|n_{4}\left|\right|T_{3}\left|\right|T_{4})$. CS selects the new temporary identities $TID_{i}^{new}$ and $TID_{j}^{new}$ for $U_{i}$ and $WD_{j}$, and then changes $\left(TID_{i}^{old}=TID_{U},TID_{i}^{new}=TID_{i}^{new}\right)$ and $\left(TID_{j}^{old}=TID_{WD},TID_{j}^{new}=TID_{j}^{new}\right)$ in its database. Then, CS calculates $TID_{i}^{*}=TID_{i}^{new}⊕h(TID_{U}\left|\right|n_{4}||SK_{CS-U})$ and $TID_{j}^{*}=TID_{j}^{new}⊕h(TID_{WD}\left|\right|n_{4}||SK_{WD-U})$ and transmits $Msg_{4}=\left\{M_{8},M_{9},M_{10},M_{11},TID_{i}^{*},TID_{j}^{*},T_{4}\right\}$ to $MD_{i}$ over an insecure channel.
**LAP-7:** 
$MD_{i}$ verifies the freshness of $|T_{5}-T_{4}|\;\leq \;\Delta T_{i}$. If it matches, $U_{i}$ calculates $n_{4}=M_{8}⊕h(TID_{U}\left|\right|n_{3}\left|\right|T_{4})$, $M_{7}^{*}=B_{i}^{*}⊕T_{4}=E_{i}⊕h\left(TID_{U}⊕HPW_{i}⊕RN_{i}\right)⊕T_{4}$, $SK_{CS-U}^{*}=h(TID_{U}\left|\right|M_{7}^{*}\left|\right|n_{3}\left|\right|n_{4}\left|\right|T_{3}\left|\right|T_{4})$, $SK_{WD-U}^{*}=M_{11}⊕h(B_{i}\left|\right|n_{1}\left|\right|n_{3}\left|\right|n_{4}\left|\right|T_{3}\left|\right|T_{4})$, $M_{9}^{*}=h(M_{4}\left|\right|M_{8}||SK_{CS-U}^{*}\left|\right|T_{3}\left|\right|T_{4})$, and $M_{10}^{*}=h(M_{4}\left|\right|M_{8}||SK_{WD-U}^{*}\left|\right|T_{3}\left|\right|T_{4})$, and then checks whether $M_{9}^{*}\overset{?}{=}M_{9}$ and $M_{10}^{*}\overset{?}{=}M_{10}$. If they are valid, $MD_{i}$ authenticates CS. After that, $MD_{i}$ stores the session keys, $SK_{CS-U}^{*}$ and $SK_{WD-U}^{*}$, and the new temporary identity, $TID_{i}^{new}=TID_{i}^{*}⊕h(TID_{U}\left|\right|n_{4}||SK_{CS-U}^{*})$.
**LAP-8:** 
$MD_{i}$ selects $n_{5}$ and computes $M_{12}=n_{4}⊕h(TID_{WD}\left|\right|n_{1}\left|\right|T_{4})$ to transmit the secret parameters securely, $M_{13}=n_{5}⊕h(TID_{WD}\left|\right|n_{1}\left|\right|T_{5})$ to transmit the secret parameters securely, $M_{14}=h(TID_{U}||SK_{WD-U}\left|\right|n_{5}\left|\right|T_{5})$ to verify the authorized entity, and then transmits $Msg_{5}=\left\{TID_{U},TID_{j}^{*},M_{12},M_{13},M_{14},T_{4},T_{5}\right\}$ to $WD_{j}$.
**LAP-9:** 
$WD_{j}$ verifies the freshness $|T_{6}-T_{5}|\;\leq \;\Delta T_{i}$. If it matches, $WD_{j}$ computes $n_{4}=M_{12}⊕h(TID_{WD}\left|\right|n_{1}\left|\right|T_{4})$, $n_{5}=M_{13}⊕h(TID_{WD}\left|\right|n_{1}\left|\right|T_{5})$, $SK_{WD-U}^{*}=h(TID_{U}||TID_{WD}||h(ID_{WD}\left||MK)|\right|n_{2}\left|\right|n_{4}\left|\right|T_{4})$, and $M_{14}^{*}=h(TID_{U}||SK_{WD-U}\left|\right|n_{5}\left|\right|T_{5})$, and then checks whether $M_{14}^{*}\overset{?}{=}M_{14}$. If it matches, $WD_{j}$ authenticates $U_{i}$ successfully. Finally, $WD_{j}$ stores a session key $SK_{WD-U}^{*}$ and a new temporary identity $TID_{j}^{*}=TID_{j}^{new}⊕h(TID_{WD}\left|\right|n_{4}||SK_{WD-U}^{*})$.

## 5. Security Flaws of Guo et al.’s Scheme

In this section, we prove that Guo et al.’s scheme [8] is not protected against the lethal security threats and cannot offer several security functionalities.

### 5.1. Impersonation Attack

According to Section 3.1, A can extract the secret credentials $\left\{PID_{WD},TID_{WD}\right\}$ stored in $WD_{j}$. Moreover, A can intercept, block, modify, replay, and delete the exchanged messages over an insecure channel. In this attack, A attempts to impersonate a legitimate entity.

- **Step 1:**A first calculates $n_{2}=M_{1}⊕h(TID_{WD}||PID_{WD})$ and a new random nonce $n_{2}^{A}$. After that, A computes $M_{1}^{A}=h(TID_{WD}||PID_{WD})⊕n_{2}^{A}$ and $M_{2}^{A}=h(M_{1}^{A}\left|\right|T_{2}\left|\right|n_{1}\left|\right|n_{2}^{A})$. After that, A transmits the message $\left\{M_{1}^{A},M_{2}^{A},TID_{WD},T_{2}\right\}$ to CS via $MD_{i}$.
- **Step 2:** After receiving the message, CS retrieves $TID_{WD}$ in its database and then obtains $\left\{ID_{WD}\right\}$, corresponding to $TID_{WD}$ in its database. Then, CS calculates $PID_{WD}=h(ID_{WD}\left||MK\right)$, $n_{2}^{A*}=h(TID_{WD}||PID_{WD})⊕M_{1}^{A}$, $n_{1}^{*}=h\left(TID_{U}⊕TID_{WD}\right)⊕M_{5}$, and $M_{2}^{A*}=h(M_{1}^{A}\left|\right|T_{2}\left|\right|n_{1}^{*}\left|\right|n_{2}^{A*})$, and checks $M_{2}^{A*}\overset{?}{=}M_{2}^{A}$. If it matches, CS authenticates A, successfully.
- **Step 3:**CS generates a random nonce $n_{4}$ and timestamp $T_{4}$. After that, CS computes $M_{7}=B_{i}⊕T_{4}=h(TID_{U}\left|\right|r_{i}\left||MK\right)⊕T_{4}$, $M_{8}=n_{4}⊕h(TID_{U}\left|\right|r_{i}\left|\right|n_{3}\left|\right|T_{4})$, $SK_{CS-U}=h(TID_{U}\left|\right|M_{7}\left|\right|n_{3}\left|\right|n_{4}\left|\right|T_{3}\left|\right|T_{4})$, $M_{9}=h(M_{4}\left|\right|M_{8}||SK_{CS-U}\left|\right|T_{3}\left|\right|T_{4})$, $SK_{WD-U}^{A}=h(TID_{U}||TID_{WD}||h(ID_{WD}\left||MK)|\right|n_{2}^{A}\left|\right|n_{4}\left|\right|T_{4})$, $M_{10}^{A}=h(M_{4}\left|\right|M_{8}||SK_{WD-U}^{A}\left|\right|T_{3}\left|\right|T_{4})$, and $M_{11}^{A}=SK_{WD-U}^{A}⊕h(B_{i}\left|\right|n_{1}\left|\right|n_{3}\left|\right|n_{4}\left|\right|T_{3}\left|\right|T_{4})$. CS selects the new temporary identities, $TID_{i}^{new}$ and $TID_{j}^{new}$ for $U_{i}$ and $WD_{j}$, and then changes $\left(TID_{i}^{old}=TID_{U},TID_{i}^{new}=TID_{i}^{new}\right)$, and $\left(TID_{j}^{old}=TID_{WD},TID_{j}^{new}=TID_{j}^{new}\right)$ in its database. Then, CS calculates $TID_{i}^{*}=TID_{i}^{new}⊕h(TID_{U}\left|\right|n_{4}||SK_{CS-U})$ and $TID_{j}^{A*}=TID_{j}^{new}⊕h(TID_{WD}\left|\right|n_{4}||SK_{WD-U}^{A})$ and transmits $\left\{M_{8},M_{9},M_{10}^{A},M_{11}^{A},TID_{i}^{*},TID_{j}^{A*},T_{4}\right\}$ to $MD_{i}$ over an open channel.
- **Step 4:** Upon receiving the message, $MD_{i}$ verifies the freshness of $|T_{5}-T_{4}|\;\leq \;\Delta T_{i}$. If it matches, $U_{i}$ calculates $n_{4}=M_{8}⊕h(TID_{U}\left|\right|n_{3}\left|\right|T_{4})$, $M_{7}^{*}=B_{i}^{*}⊕T_{4}=E_{i}⊕h\left(TID_{U}⊕HPW_{i}⊕RN_{i}\right)⊕T_{4}$, $SK_{CS-U}^{*}=h(TID_{U}\left|\right|M_{7}^{*}\left|\right|n_{3}\left|\right|n_{4}\left|\right|T_{3}\left|\right|T_{4})$, $SK_{WD-U}^{A*}=M_{11}^{A}⊕h(B_{i}\left|\right|n_{1}\left|\right|n_{3}\left|\right|n_{4}\left|\right|T_{3}\left|\right|T_{4})$, $M_{9}^{*}=h(M_{4}\left|\right|M_{8}||SK_{CS-U}^{*}\left|\right|T_{3}\left|\right|T_{4})$, and $M_{10}^{A*}=h(M_{4}\left|\right|M_{8}||SK_{WD-U}^{A*}\left|\right|T_{3}\left|\right|T_{4})$, and then checks whether $M_{9}^{*}\overset{?}{=}M_{9}$ and $M_{10}^{A*}\overset{?}{=}M_{10}^{A}$. If they are valid, $MD_{i}$ authenticates CS. After that, $MD_{i}$ stores the session keys $SK_{CS-U}^{*}$ and $SK_{WD-U}^{A*}$ and the new temporary identity $TID_{i}^{new}=TID_{i}^{*}⊕h(TID_{U}\left|\right|n_{4}||SK_{CS-U}^{*})$.
- **Step 5:** Then, $MD_{i}$ selects $n_{5}$ and computes $M_{12}=n_{4}⊕h(TID_{WD}\left|\right|n_{1}\left|\right|T_{4})$, $M_{13}=n_{5}⊕h(TID_{WD}\left|\right|n_{1}\left|\right|T_{5})$, $M_{14}^{A}=h(TID_{U}||SK_{WD-U}^{A}\left|\right|n_{5}\left|\right|T_{5})$, and then transmits $\left\{TID_{U},TID_{j}^{A*},M_{12},M_{13},M_{14}^{A},T_{4},T_{5}\right\}$ to $WD_{j}$.
- **Step 6:** After eavesdropping on the message, $\left\{TID_{U},TID_{j}^{A*},M_{12},M_{13},M_{14}^{A},T_{4},T_{5}\right\}$, A calculates $n_{4}=M_{12}⊕h(TID_{WD}\left|\right|n_{1}\left|\right|T_{4})$, $n_{5}=M_{13}⊕h(TID_{WD}\left|\right|n_{1}\left|\right|T_{5})$, $SK_{WD-U}^{A*}=h(TID_{U}||TID_{WD}||h(ID_{WD}\left||MK)|\right|n_{2}^{A}\left|\right|n_{4}\left|\right|T_{4})$, and $M_{14}^{A*}=h(TID_{U}||SK_{WD-U}^{A}\left|\right|n_{5}\left|\right|T_{5})$. Note that $h(ID_{WD}||MK)$, included in the session key, is the same as $PID_{WD}$. Finally, A stores a session key $SK_{WD-U}^{A*}$ and a new temporary identity $TID_{j}^{A*}=TID_{j}^{new}⊕h(TID_{WD}\left|\right|n_{4}||SK_{WD-U}^{A*})$.

Consequently, their scheme is not resistant to impersonation attacks since A can impersonate the legitimate $WD_{j}$.

### 5.2. MITM Attack

Based on the threat model, A can extract the secret parameters $\left\{PID_{WD},TID_{WD}\right\}$ stored in $WD_{j}$. Furthermore, A can block, intercept, modify, replay, and delete the transmitted messages via an open channel.

- **Step 1:** After eavesdropping on the message $\left\{n_{1},T_{1}\right\}$ via a public channel, A first calculates $n_{2}=M_{1}⊕h(TID_{WD}||PID_{WD})$ and $M_{2}=h(M_{1}\left|\right|T_{2}\left|\right|n_{1}\left|\right|n_{2})$. After that, A transmits $\left\{M_{1},M_{2},TID_{WD},T_{2}\right\}$.
- **Step 2:** After eavesdropping on the message $\left\{TID_{U},TID_{j}^{*},M_{12},M_{13},M_{14},T_{4},T_{5}\right\}$ via a public channel, A computes $n_{4}=M_{12}⊕h(TID_{WD}\left|\right|n_{1}\left|\right|T_{4})$ and $n_{5}=M_{13}⊕h(TID_{WD}\left|\right|n_{1}\left|\right|T_{5})$.
- **Step 3:**A calculates a session key $SK_{WD-U}^{*}=h(TID_{U}||TID_{WD}||h(ID_{WD}\left||MK)|\right|n_{2}\left|\right|n_{4}\left|\right|T_{4})$, where $h(ID_{WD}||MK)$, included in the session key, is the same as $PID_{WD}$. Finally, A successfully calculates $M_{14}^{*}=h(TID_{U}||SK_{WD-U}\left|\right|n_{5}\left|\right|T_{5})$ and then verifies $M_{14}^{*}\overset{?}{=}M_{14}$. Hence, their scheme is not protected against this attack.

### 5.3. Session Key Disclosure Attack

Based on Section 5.2, A extracts $n_{4}=M_{12}⊕h(TID_{WD}\left|\right|n_{1}\left|\right|T_{4})$ and $n_{5}=M_{13}⊕h(TID_{WD}\left|\right|n_{1}\left|\right|T_{5})$, and then computes a session key $SK_{WD-U}^{*}=h(TID_{U}||TID_{WD}||h(ID_{WD}\left||MK)|\right|n_{2}\left|\right|n_{4}\left|\right|T_{4})$ successfully. As a result, A can successfully obtain a common session key $SK_{WD-U}^{*}$ between legitimate $U_{i}$ and $WD_{j}$. Thus, Guo et al.’s scheme is insecure to this attack.

### 5.4. Mutual Authentication

In Guo et al.’s scheme, they claimed to provide mutual authentication between the entities. Unfortunately, according to Section 5.2 and Section 5.3, A can successfully generate the sensitive messages, $M_{10}=h(M_{4}\left|\right|M_{8}||SK_{WD-U}\left|\right|T_{3}\left|\right|T_{4})$ and $M_{14}=h(TID_{U}||SK_{WD-U}^{A}\left|\right|n_{5}\left|\right|T_{5})$, for mutual authentication. Thus, Guo et al.’s scheme cannot guarantee secure mutual authentication between the legitimate $U_{i}$ and $WD_{j}$.

### 5.5. Untraceability

Guo et al. claimed that their protocol achieved untraceability. However, according to Section 5.2 and Section 5.3, A calculates the random nonces $n_{4}=M_{12}⊕h(TID_{WD}\left|\right|n_{1}\left|\right|T_{4})$ and $n_{5}=M_{13}⊕h(TID_{WD}\left|\right|n_{1}\left|\right|T_{5})$ and then computes a session key $SK_{WD-U}^{*}=h(TID_{U}||TID_{WD}||h(ID_{WD}\left||MK)|\right|n_{2}\left|\right|n_{4}\left|\right|T_{4})$. After that, A successfully calculates a new temporary identity $TID_{j}^{new}=TID_{j}^{*}⊕h(TID_{WD}\left|\right|n_{4}||SK_{WD-U}^{*})$. Thus, Guo et al.’s scheme does not achieve untraceability because A can trace the authorized $WD_{j}$ through their new temporary identity.

## 6. Proposed Scheme

**Figure 3:**
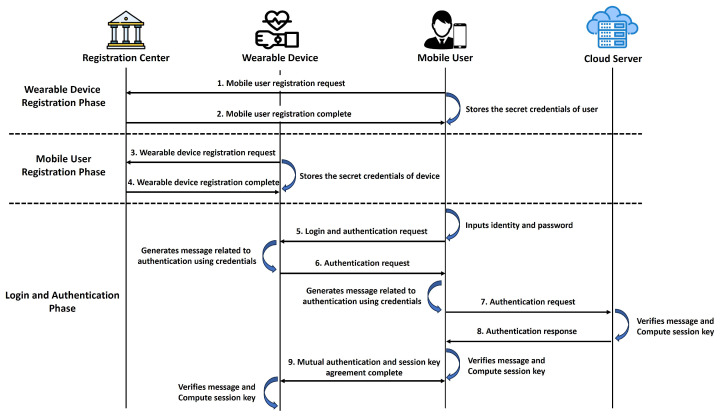
The overall flowchart during the AKA phase.

The existing related schemes for wearable computing are not protected against potential security attacks. Thus, we propose a “robust and efficient authentication and group–proof scheme using the PUF for wearable computing (AGPS-PUF)” to improve the security flaws of the existing schemes. The AGPS-PUF is resilient to cyber/physical security attacks and provides necessary security functionalities. The AGPS-PUF consists of six phases: (1) system setup, (2) registration, (3) login and authentication, (4) group proof, and (5) password update. We show the overall flowchart during the AKA phase of the AGPS-PUF, as shown in Figure 3.

### 6.1. System Setup Phase

In this section, RC first sets the secret credentials for each $WD_{j}$. The following are detailed descriptions:

**SP-1:** 
RC selects a master private key MK for CS.
**SP-2:** 
RC chooses a unique identity $ID_{WD}$ for each $WD_{j}$ and then generates a temporary identity $TID_{WD}$ for each $WD_{j}$.
**SP-3:** 
RC stores $\left\{ID_{WD},TID_{WD}\right\}$ in CS’s secure database and then stores the secret credentials $\left\{ID_{WD},TID_{WD},h\left(\cdot \right)\right\}$ in the memory of $WD_{j}$.

### 6.2. Registration Phase

This phase consists of two parts: $U_{i}$ and $WD_{j}$ registration phases.

#### 6.2.1. User Registration Phase

In this phase, $U_{i}$ registers within RC and then obtains the secret credentials from RC.

**URP-1:** 
$U_{i}$ chooses unique $ID_{U}$ and $PW_{U}$ in $MD_{i}$. After that, $MD_{i}$ selects a random number $a_{i}$ and generates a set of $\left(C_{U}^{x},R_{U}^{x}\right)$ based on the PUF to ensure the unique physical properties of the device. Then, $MD_{i}$ computes $HID_{U}=h(ID_{U}\left|\right|a_{i})$ and $HPW_{i}=h(PW_{U}\left|\right|a_{i}\left|\right|R_{U}^{x})$ and then transmits $\left\{HID_{U},HPW_{i},\left(C_{U}^{x},R_{U}^{x}\right)\right\}$ to RC over a secure channel.
**URP-2:** 
RC generates a temporary identity $TID_{U}$ and computes $X_{i}=h(TID_{U}\left||MK|\right|R_{U}^{x})$, $X_{UW}=h(ID_{RC}\left||MK\right)$, $A_{i}=(X_{i}\left|\right|X_{UW})⊕h(HID_{U}⊕h(HPW_{i}\left|\right|R_{U}^{x}))$, $B_{i}=h(HPW_{i}||TID_{U}\left|\right|X_{i})$, $C_{i}=X_{i}⊕h(ID_{CS}||TID_{U}\left||MK\right)$, Finally, RC stores $\left\{TID_{U},\left(C_{U}^{x},R_{U}^{x}\right),C_{i}\right\}$ in CS’s database and then sends $\left\{A_{i},B_{i},TID_{U}\right\}$ to $MD_{i}$ over a secure channel.
**URP-3:** 
Finally, $MD_{i}$ computes $D_{i}=a_{i}⊕h(ID_{U}||PW_{U}\left|\right|R_{U}^{x})$ and stores $\left\{A_{i},B_{i},D_{i},TID_{U}\right\}$ in its memory.

#### 6.2.2. Wearable Device Registration Phase

In this phase, $WD_{j}$ registers within RC and then obtains the secret credentials from RC.

**WDRP 1:** 
$WD_{j}$ generates a random number $b_{j}$ and a set $\left(C_{WD}^{x},R_{WD}^{x}\right)$ under the PUF to ensure the unique physical properties of the device. After that, $WD_{j}$ calculates $Q_{j}=b_{j}⊕h(ID_{WD}\left|\right|R_{WD}^{x})$ and $W_{j}=h(ID_{WD}\left|\right|b_{j}\left|\right|R_{WD}^{x})$. After that, $WD_{j}$ sends $\left\{TID_{WD},Q_{j},W_{j},\left(C_{WD}^{x},R_{WD}^{x}\right)\right\}$ to RC.
**WDRP 2:** 
RC retrieves the corresponding $ID_{WD}$ stored in the database using $TID_{WD}$. After that, RC computes $b_{j}^{*}=Q_{j}⊕h(ID_{WD}\left|\right|R_{WD}^{x})$, and $W_{j}^{*}=h(ID_{WD}^{*}\left|\right|b_{j}^{*}\left|\right|R_{WD}^{x})$, and verifies $W_{j}^{*}\overset{?}{=}W_{j}$. If it is invalid, CS terminates $WD_{j}$’s registration request; otherwise, RC computes $PID_{WD}=h(TID_{WD}\left||MK\right)$, $Z_{j}=h(TID_{WD}\left||MK|\right|R_{WD}^{x})$, $X_{UW}=h(ID_{RC}\left||MK\right)$, $E_{j}=(X_{UW}\left|\right|Z_{j}||PID_{WD})⊕h(ID_{WD}||TID_{WD}\left|\right|R_{WD}^{x}\left|\right|b_{j})$, and $Y_{j}=Z_{j}⊕h(ID_{CS}||TID_{WD}\left||MK\right)$. After that, RC stores $\left\{Y_{j},\left(C_{WD}^{x},R_{WD}^{x}\right)\right\}$ in CS’s secure database and then transmits $\left\{E_{j}\right\}$ to $WD_{j}$.
**WDRP 3:** 
Finally, $WD_{j}$ computes $O_{j}=b_{j}⊕h\left(R_{WD}^{x}⊕TID_{WD}⊕ID_{WD}\right)$ and then stores $\left\{E_{j},O_{j}\right\}$ in memory.

### 6.3. Login and Authentication Phase

**Figure 4:**
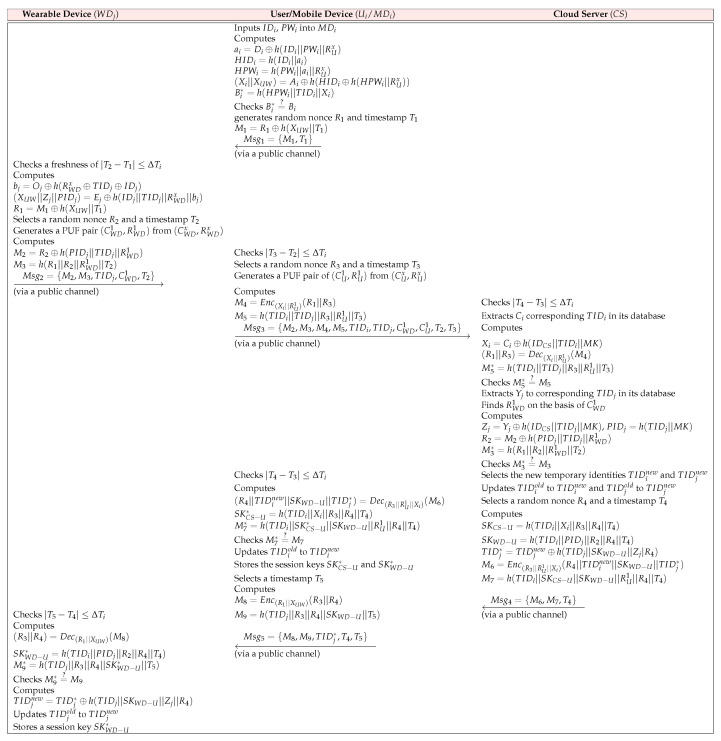
Login and Authentication Phase of the AGPS-PUF.

The registered $U_{i}$ and $WD_{j}$ should establish a common session key with the help of CS to use reliable medical services. This phase is illustrated in Figure 4.

**LAP-1:** 
$U_{i}$ first inputs a unique identity $ID_{U}$ and password $PW_{i}$ into $MD_{i}$. After that, $MD_{i}$ calculates $a_{i}=D_{i}⊕h(ID_{U}||PW_{i}\left|\right|R_{U}^{x})$, $HID_{U}=h(ID_{U}\left|\right|a_{i})$, $HPW_{i}=h(PW_{i}\left|\right|a_{i}\left|\right|R_{U}^{x})$, $(X_{i}\left|\right|X_{UW})=A_{i}⊕h(HID_{U}⊕h(HPW_{i}\left|\right|R_{U}^{x}))$, and $B_{i}^{*}=h(HPW_{i}||TID_{U}\left|\right|X_{i})$ and then checks $B_{i}^{*}\overset{?}{=}B_{i}$. If it matches, $MD_{i}$ generates a random nonce $R_{1}$ and a timestamp $T_{1}$. Then, $MD_{i}$ computes $M_{1}=R_{1}⊕h(X_{UW}\left|\right|T_{1})$ to make the masked random nonce and transmits $Msg_{1}=\left\{M_{1},T_{1}\right\}$ to $WD_{j}$ via an insecure channel.
**LAP-2:** 
$WD_{j}$ checks the freshness of $|T_{2}-T_{1}|\;\leq \;\Delta T_{i}$, where $T_{2}$ is the current timestamp and $\Delta T_{1}$ is the maximum transmission delay for the message to be transmitted between $MD_{i}$ and $WD_{j}$. If it matches, $WD_{j}$ calculates $b_{j}=O_{j}⊕h\left(R_{WD}^{x}⊕TID_{WD}⊕ID_{WD}\right)$, $(X_{UW}\left|\right|Z_{j}||PID_{WD})=E_{j}⊕h(ID_{WD}||TID_{WD}\left|\right|R_{WD}^{x}\left|\right|b_{j})$, and $R_{1}=M_{1}⊕h(X_{UW}\left|\right|T_{1})$. Then, $WD_{j}$ selects $R_{2}$ and $T_{2}$. After that, $WD_{j}$ chooses a pair of $\left(C_{WD}^{1},R_{WD}^{1}\right)$ from the preloaded CRPs $\left(C_{WD}^{x},R_{WD}^{x}\right)$ to ensure the unique physical properties of the device and computes $M_{2}=R_{2}⊕h(PID_{WD}||TID_{WD}\left|\right|R_{WD}^{1})$ to make the masked random nonce, and $M_{3}=h(R_{1}\left|\right|R_{2}\left|\right|R_{WD}^{1}\left|\right|T_{2})$ to verify the authorized entity, and then transmits $Msg_{2}=\left\{M_{2},M_{3},TID_{WD},C_{WD}^{1},T_{2}\right\}$ to $MD_{i}$.
**LAP-3:** 
After receiving the message, $MD_{i}$ verifies the freshness of $|T_{3}-T_{2}|\;\leq \;\Delta T_{i}$. If it matches, $MD_{i}$ generates $R_{3}$ and a timestamp $T_{3}$ and chooses a pair of $\left(C_{U}^{1},R_{U}^{1}\right)$ from the preloaded CRPs $\left(C_{U}^{x},R_{U}^{x}\right)$ to ensure the unique physical properties of the device. After that, $MD_{i}$ decrypts $M_{4}=Enc_{\left(X_{i}||R_{U}^{1}\right)}(R_{1}\left|\right|R_{3})$ to obtain the random nonce and calculates $M_{5}=h(TID_{U}||TID_{WD}\left|\right|R_{3}\left|\right|R_{U}^{1}\left|\right|T_{3})$ to verify the authorized entity, and then transmits $Msg_{3}=\left\{M_{2},M_{3},M_{4},M_{5},TID_{U},TID_{WD},C_{WD}^{1},C_{U}^{1},T_{2},T_{3}\right\}$ to CS through a public channel.
**LAP-4:** 
After receiving $Msg_{3}$ from $MD_{i}$, CS checks the freshness of $|T_{4}-T_{3}|\;\leq \;\Delta T_{i}$. If it matches, CS finds $R_{U}^{1}$ on the basis of $C_{U}^{1}$. After that, CS extracts $C_{i}$ corresponding to $TID_{U}$ in its database. CS decrypts $(R_{1}\left|\right|R_{3})=Dec_{\left(X_{i}||R_{U}^{1}\right)}\left(M_{4}\right)$, and computes $X_{i}=C_{i}⊕h(ID_{CS}||TID_{U}\left||MK\right)$, and $M_{5}^{*}=h(TID_{U}||TID_{WD}\left|\right|R_{3}\left|\right|R_{U}^{1}\left|\right|T_{3})$, and verifies $M_{5}^{*}\overset{?}{=}M_{5}$. If it matches, CS aborts the current session; otherwise, CS extracts $Y_{j}$ to the corresponding $TID_{WD}$ in its database. Then, CS finds $R_{WD}^{1}$ on the basis of $C_{WD}^{1}$ and computes $Z_{j}=Y_{j}⊕h(ID_{CS}||TID_{WD}\left||MK\right)$, $PID_{WD}=h(TID_{WD}\left||MK\right)$, $R_{2}=M_{2}⊕h(PID_{WD}||TID_{WD}\left|\right|R_{WD}^{1})$, and $M_{3}^{*}=h(R_{1}\left|\right|R_{2}\left|\right|R_{WD}^{1}\left|\right|T_{2})$, and verifies $M_{3}^{*}\overset{?}{=}M_{3}$. If it matches, CS selects the new temporary identities, $TID_{i}^{new}$ and $TID_{j}^{new}$ for $U_{i}$ and $WD_{j}$, and updates $TID_{i}^{old}$ to $TID_{i}^{new}$ and $TID_{j}^{old}$ to $TID_{j}^{new}$ in its database. CS generates $R_{4}$ and $T_{4}$ and computes $SK_{CS-U}=h(TID_{U}\left|\right|X_{i}\left|\right|R_{3}\left|\right|R_{4}\left|\right|T_{4})$, $SK_{WD-U}=h(TID_{U}||PID_{WD}\left|\right|R_{2}\left|\right|R_{4}\left|\right|T_{4})$, $TID_{j}^{*}=TID_{j}^{new}⊕h(TID_{WD}||SK_{WD-U}\left|\right|Z_{j}\left|R_{4}\right)$, $M_{6}=Enc_{\left(R_{3}||R_{U}^{1}||X_{i}\right)}(R_{4}||TID_{i}^{new}||SK_{WD-U}||TID_{j}^{*})$ to transmit the secret parameters securely, and $M_{7}=h(TID_{U}||SK_{CS-U}||SK_{WD-U}\left|\right|R_{U}^{1}\left|\right|R_{4}\left|\right|T_{4})$ to verify the authorized entity. Finally, CS sends $Msg_{4}=\left\{M_{6},M_{7},T_{4}\right\}$ to $MD_{i}$.
**LAP-5:** 
$MD_{i}$ verifies the freshness of $|T_{4}-T_{3}|\;\leq \;\Delta T_{i}$. If it matches, $MD_{i}$ decrypts $(R_{4}||TID_{i}^{new}||SK_{WD-U}||TID_{j}^{*})=Dec_{\left(R_{3}||R_{U}^{1}||X_{i}\right)}\left(M_{6}\right)$ and computes $SK_{CS-U}^{*}=h(TID_{U}\left|\right|X_{i}\left|\right|R_{3}\left|\right|R_{4}\left|\right|T_{4})$, and $M_{7}^{*}=h(TID_{U}||SK_{CS-U}^{*}||SK_{WD-U}\left|\right|R_{U}^{1}\left|\right|R_{4}\left|\right|T_{4})$, and verifies $M_{7}^{*}\overset{?}{=}M_{7}$. If it is not equal, $MD_{i}$ terminates the current session; otherwise, $MD_{i}$ updates a new temporary identity $TID_{i}^{old}$ to $TID_{i}^{new}$ and stores the session keys $SK_{CS-U}^{*}$ and $SK_{WD-U}^{*}$. After that, $MD_{i}$ generates a timestamp $T_{5}$ and computes $M_{8}=Enc_{\left(R_{1}||X_{UW}\right)}(R_{3}\left|\right|R_{4})$ to transmit the secret parameters securely, and $M_{9}=h(TID_{WD}\left|\right|R_{3}\left|\right|R_{4}||SK_{WD-U}\left|\right|T_{5})$ to verify the authorized entity, and then transmits $Msg_{5}=\left\{M_{8},M_{9},TID_{j}^{*},T_{4},T_{5}\right\}$ to $WD_{j}$ over a public channel.
**LAP-6:** 
$WD_{j}$ checks the freshness of $|T_{5}-T_{4}|\;\leq \;\Delta T_{i}$. If it matches, $WD_{j}$ computes $(R_{3}\left|\right|R_{4})=Dec_{\left(R_{1}||X_{UW}\right)}\left(M_{8}\right)$, $SK_{WD-U}^{*}=h(TID_{U}||PID_{WD}\left|\right|R_{2}\left|\right|R_{4}\left|\right|T_{4})$, and $M_{9}^{*}=h(TID_{WD}\left|\right|R_{3}\left|\right|R_{4}||SK_{WD-U}^{*}\left|\right|T_{5})$, and verifies $M_{9}^{*}\overset{?}{=}M_{9}$. If it matches, $WD_{j}$ authenticates $U_{i}$, successfully and then calculates $TID_{j}^{new}=TID_{j}^{*}⊕h(TID_{WD}||SK_{WD-U}\left|\right|Z_{j}\left|\right|R_{4})$. Finally, $WD_{j}$ updates a new temporary identity $TID_{j}^{old}$ to $TID_{j}^{new}$ and stores a session key $SK_{WD-U}^{*}$.

### 6.4. Group–Proof Generation and Verification Phases

**Figure 5:**
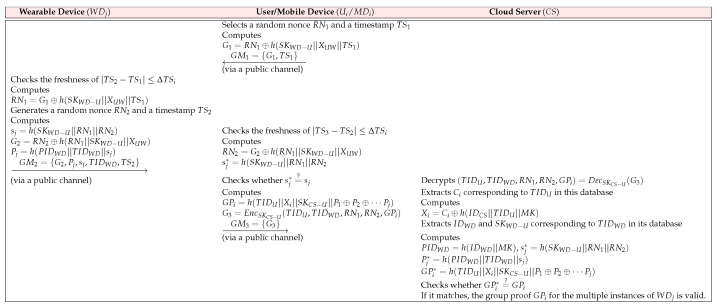
Group–proof generation and verification phase of the AGPS-PUF.

After the authentication process is executed successfully, $U_{i}$ generates a group proof for multiple $WD_{j}$ by $MD_{i}$, indicating that $WD_{j}$ belongs to the same $U_{i}$ and then sends the group proof to CS for verification. This phase is illustrated in Figure 5.

**GPGV 1:** 
$MD_{i}$ for authorized $U_{i}$ selects $RN_{1}$ and $TS_{1}$. After that, $MD_{i}$ computes $G_{1}=RN_{1}⊕h(SK_{WD-U}\left|\right|X_{UW}||TS_{1})$ and then sends $GM_{1}=\left\{G_{1},TS_{1}\right\}$ to $WD_{j}$ over a public channel.
**GPGV 2:** 
$WD_{j}$ verifies the freshness of $|TS_{2}-TS_{1}|\;\leq \;\Delta TS_{i}$. If it matches, $WD_{j}$ calculates $RN_{1}=G_{1}⊕h(SK_{WD-U}\left|\right|X_{UW}||TS_{1})$ and generates a random nonce $RN_{2}$ and a timestamp $TS_{2}$. After that, $WD_{j}$ computes $s_{j}=h(SK_{WD-U}||RN_{1}||RN_{2})$, $G_{2}=RN_{2}⊕h(RN_{1}||SK_{WD-U}\left|\right|X_{UW})$, and $P_{j}=h(PID_{WD}||TID_{WD}\left|\right|s_{j})$, and then transmits $GM_{2}=\left\{G_{2},P_{j},s_{j},TID_{WD},TS_{2}\right\}$ to $MD_{i}$.
**GPGV 3:** 
$MD_{i}$ checks the freshness of $|TS_{3}-TS_{2}|\;\leq \;\Delta TS_{i}$. If it matches, $MD_{i}$ computes $RN_{2}=G_{2}⊕h(RN_{1}||SK_{WD-U}\left|\right|X_{UW})$ and $s_{j}^{*}=h(SK_{WD-U}||RN_{1}\left|\right|RN_{2})$, and checks whether $s_{j}^{*}\overset{?}{=}s_{j}$. If it matches, $MD_{i}$ generates the group proof $GP_{i}=h(TID_{U}\left|\right|X_{i}||SK_{CS-U}\left|\right|P_{1}⊕P_{2}⊕\cdots P_{j})$ for all wearable devices. Finally, $WD_{j}$ encrypts $G_{3}=Enc_{SK_{CS-U}}\left(TID_{U},TID_{WD},RN_{1},RN_{2},GP_{i}\right)$ using a session key $SK_{CS-U}$ and then transmits $GM_{3}=\left\{G_{3}\right\}$ to CS over an open channel.
**GPGV 4:** 
CS decrypts $\left(TID_{U},TID_{WD},RN_{1},RN_{2},GP_{i}\right)=Dec_{SK_{CS-U}}\left(G_{3}\right)$ using a session key $SK_{CS-U}$. CS extracts $C_{i}$ corresponding to $TID_{U}$ in this database, computes $X_{i}=C_{i}⊕h(ID_{CS}||TID_{U}\left||MK\right)$, and then extracts $ID_{WD}$ and $SK_{WD-U}$, corresponding to $TID_{WD}$ in its database. After that, CS computes $PID_{WD}=h(ID_{WD}\left||MK\right)$, $s_{j}^{*}=h(SK_{WD-U}||RN_{1}||RN_{2})$, $P_{j}^{*}=h(PID_{WD}||TID_{WD}\left|\right|s_{j})$, and $GP_{i}^{*}=h(TID_{U}\left|\right|X_{i}||SK_{CS-U}\left|\right|P_{1}⊕P_{2}⊕\cdots P_{j})$ and then checks whether $GP_{i}^{*}\overset{?}{=}GP_{i}$. If it matches, CS successfully verifies that the multiple instances of $WD_{j}$ belong to the same $U_{i}$ through the group proof.

### 6.5. Password Update Phase

If $U_{i}$ wishes to obtain a new $PW_{i}$, $U_{i}$ can freely update their old $PW_{i}$ without interacting with RC.

**PUP-1:** 
$U_{i}$ inputs $ID_{U}$ and an old password $PW_{i}^{old}$ in $MD_{i}$.
**PUP-2:** 
$MD_{i}$ chooses a set of $\left(C_{U}^{x},R_{U}^{x}\right)$, and computes $a_{i}=D_{i}⊕h(ID_{U}||PW_{i}\left|\right|R_{U}^{x})$, $HID_{U}=h(ID_{U}\left|\right|a_{i})$, $HPW_{i}=h(PW_{i}\left|\right|a_{i}\left|\right|R_{U}^{x})$, $(X_{i}\left|\right|X_{UW})=A_{i}⊕h(HID_{U}⊕h(HPW_{i}\left|\right|R_{U}^{x})$, and $B_{i}^{*}=h(HID_{U}||TID_{U}\left|\right|X_{i})$, and verifies whether $B_{i}^{*}\overset{?}{=}B_{i}$. If the condition is met, $U_{i}$ is prompted to choose a new password.
**PUP-3:** 
$U_{i}$ inputs a new $PW_{i}^{new}$ and computes $HPW_{i}^{new}=h(PW_{i}^{new}\left|\right|a_{i}\left|\right|R_{U}^{x})$, $A_{i}^{new}=(X_{i}\left|\right|X_{UW})⊕h(HID_{U}⊕h(HPW_{i}^{new}\left|\right|R_{U}^{x}))$, $B_{i}^{new}=h(HPW_{i}^{new}||TID_{U}\left|\right|X_{i})$, $D_{i}^{new}=a_{i}⊕h(ID_{U}||PW_{i}^{new}\left|\right|R_{U}^{x})$. Finally, $MD_{i}$ replaces $\left\{A_{i},B_{i},D_{i}\right\}$ with $\left\{A_{i}^{new},B_{i}^{new},D_{i}^{new}\right\}$. As a result, $MD_{i}$ contains $\left\{A_{i}^{new},B_{i},D_{i}^{new},TID_{U}\right\}$.

## 7. Security Analysis

The following introduces the informal/formal security analyses.

### 7.1. Informal Security Analysis

We demonstrate that the AGPS-PUF can prevent“lethal security attacks” and allow “anonymity, untraceability, and mutual authentication”.

#### 7.1.1. Impersonation Attack

This attack means that A attempts to impersonate the legitimate user by eavesdropping on the exchanged data over an open channel. In this case, A should generate the authentication messages $\left\{Msg_{1},Msg_{2},Msg_{3}\right\}$, and $\left\{Msg_{4},Msg_{5}\right\}$. However, it is difficult to generate the sensitive messages since A cannot obtain the “random nonces $\left\{R_{1},R_{3}\right\}$” and “secret credential $\left\{X_{i},X_{UW}\right\}$”. Consequently, the AGPS-PUF is resistant to impersonation attacks because A cannot successfully generate the sensitive messages of the legitimate user.

#### 7.1.2. MITM Attack

According to Section 3.1, A can inject, modify, eavesdrop, intercept, delete, and block the exchanged messages, $\left\{Msg_{1},Msg_{2},Msg_{3},Msg_{4},Msg_{5}\right\}$, in the bidirectional communication between $U_{i}$, $WD_{j}$, and CS, and then attempt to obtain sensitive information from legitimate entities. However, A cannot generate sensitive messages since all messages are masked with the PUF responses, $\left\{R_{U}^{1},R_{WD}^{1}\right\}$, and fresh random nonces $\left\{R_{1},R_{2},R_{3}\right\}$, by using “XOR” and “hash” functions. Hence, the AGPS-PUF is secure against MITM attacks since A cannot obtain sensitive information from legitimate entities.

#### 7.1.3. Session Key Disclosure Attack

Based on the information presented in Section 3.1, A can steal the MD and then extract the secret information $\left\{A_{i},B_{i},D_{i},TID_{U}\right\}$ stored in the memory. In the AGPS-PUF, A should obtain the real identities $\left\{ID_{U},ID_{WD},ID_{CS}\right\}$ and random nonces $\left\{R_{2},R_{3},R_{4}\right\}$ to calculate the session keys, $SK_{CS-U}=h(TID_{U}\left|\right|X_{i}\left|\right|R_{3}\left|\right|R_{4}\left|\right|T_{4})$ and $SK_{WD-U}=h(TID_{U}||PID_{WD}\left|\right|R_{2}\left|\right|R_{4}\left|\right|T_{4})$. However, it is impossible for A to obtain the common session keys, $SK_{CS-U}$ and $SK_{WD-U}$, since the random nonces and real identities are preserved with secret parameters $\left\{X_{i},X_{UW},Z_{j}\right\}$, and the PUF parameters $\left\{R_{U}^{1},R_{WD}^{1}\right\}$, using cryptographic primitives. Hence, the AGPS-PUF resists session key disclosure attacks.

#### 7.1.4. Replay Attack

A eavesdrops on the transmitted messages $\left\{Msg_{1},Msg_{2},Msg_{3},Msg_{4},Msg_{5}\right\}$ during the AKA phase and then attempts to authenticate with other parties by transmitting the intercepted data in the previous session. A solution to prevent replay attacks, such as the existing schemes [37,38], is to add random nonces and timestamps to the information exchanged so that the data are unique for each authentication phase. Thus, the AGPS-PUF verifies the freshness of $T_{i}$. Moreover, the data are masked with $\left\{R_{1},R_{2},R_{3},R_{4}\right\}$. Therefore, even if A selects and sends valid authentication messages to legitimate entities, the AGPS-PUF is secure against replay attacks since the current timestamp freshness is incorrect.

#### 7.1.5. Physical Wearable Device Capture Attack

Assume that $WD_{j}$s are physically captured by A and then extract $\left\{E_{j},O_{j}\right\}$ in $WD_{j}$’s memory, where $E_{j}=(X_{UW}\left|\right|Z_{j}||PID_{WD})⊕h(ID_{WD}||TID_{WD}\left|\right|R_{WD}^{x}\left|\right|b_{j})$ and $O_{j}=b_{j}⊕h\left(R_{WD}^{x}⊕TID_{WD}⊕ID_{WD}\right)$. However, A does not successfully compute $SK_{WD-U}^{*}$ between $U_{i}$ and $WD_{j}$ without the knowledge of $\left\{R_{2},R_{4}\right\}$ and the secret credentials $\left\{X_{UW}\right\}$. In addition, the PUF pairs $\left\{\left(C_{WD}^{1},R_{WD}^{1}\right)\right\}$ are distinct, independent, and secure for all batched $WD_{j}$. Hence, the AGPS-PUF is resilient against physical wearable device capture attacks since the PUF output depends on the inherent physical fluctuations of the IC chip.

#### 7.1.6. Stolen Verifier Attack

In this attack, A extracts and learns the secret parameters related to $U_{i}$ and $WD_{j}$, which are stored in the database of CS, and it then attempts to masquerade as a legitimate entity. However, even if A obtains the stored parameters $\left\{TID_{U},\left(C_{U}^{x},R_{U}^{x}\right),C_{i}\right\}$ for $U_{i}$ and $\left\{Y_{j},\left(C_{WD}^{x},R_{WD}^{x}\right)\right\}$ for $WD_{j}$, A cannot calculate the common session keys $\left\{SK_{WD-U},SK_{CS-U}\right\}$, and impersonate a legitimate entity. Unfortunately, A does not obtain the secret credentials $\left\{C_{i},Y_{j}\right\}$ that are masked with RC’s master secret key MK by performing the cryptographic primitives. Furthermore, PUF pairs $\left(C_{U}^{x},R_{U}^{x}\right)$, and $\left(C_{WD}^{x},R_{WD}^{x}\right)$ for $U_{i}$ and $WD_{j}$ are computationally infeasible for A to derive the fresh PUF because the PUF output depends on the inherent physical fluctuations of the IC. Hence, the AGPS-PUF is resistant to stolen verifier attacks.

#### 7.1.7. Offline Password-Guessing Attack

Referring to the information presented in Section 3.1, we assume that A can intercept the transmitted information and then extract the secret credentials stored in the MD. Then, A attempts to use these attacks to guess $U_{i}$’s real $PW_{i}$. However, $PW_{i}$ is composed as $MPW_{i}=h(PW_{i}\left|\right|a_{i}\left|\right|R_{U}^{x})$. Therefore, it is impossible for A to correctly guess $PW_{i}$ without knowledge of the random number $a_{i}$ and the PUF response value $R_{U}^{x}$. As a result, the offline password-guessing attack is not feasible in the AGPS-PUF.

#### 7.1.8. Desynchronization Attack

In the AGPS-PUF, the temporary identities, $TID_{U}$ and $TID_{WD}$, are assigned to $U_{i}$ and $WD_{j}$ during the AKA phase and then tables are maintained from CS. Since both the old temporary identities, i.e., $TID_{i}^{old}$ and $TID_{j}^{old}$, are stored, if the last acknowledgment messages are blocked or lost due to time delay, there will always be consistent temporary identities between $U_{i}$, $WD_{j}$, and CS. Thus, the AGPS-PUF is resistant to desynchronization attacks.

#### 7.1.9. Privileged Insider Attack

In this attack, A is a privileged insider of the proposed system. Hence, we assume that A is able to obtain the request message $\left\{HPW_{i},HPW_{i},\left(C_{U}^{x},R_{U}^{x}\right)\right\}$ from the remote user $U_{i}$. However, the secret credentials, $\left\{X_{i},Z_{j},X_{UW}\right\}$ of $M_{i}$, and $WD_{j}$, are computationally infeasible for A without knowledge of the master private key MK and identity $ID_{CS}$. Thus, the AGPS-PUF can prevent privileged insider attacks because A cannot correctly generate the sensitive information of $U_{i}$ and $WD_{j}$.

#### 7.1.10. Mutual Authentication

In the AGPS-PUF, all participants successfully perform secure mutual authentication. After obtaining the authentication request messages, $\left\{M_{3},M_{5}\right\}$, $CS_{j}$ check whether $M_{5}^{*}\overset{?}{=}h(TID_{U}||TID_{WD}\left|\right|R_{3}\left|\right|R_{U}^{1}\left|\right|T_{3})$ to verify the authenticity and integrity of the received message. If it matches, CS is authenticated with $U_{i}$. CS then verifies whether $M_{3}^{*}\overset{?}{=}h(R_{1}\left|\right|R_{2}\left|\right|R_{WD}^{1}\left|\right|T_{2})$ to verify the authenticity and integrity of the received message. If it matches, CS is authenticated with $WD_{j}$. Upon receiving the authentication message, $\left\{M_{7}\right\}$, $U_{i}$ checks $M_{7}^{*}\overset{?}{=}h(TID_{U}||SK_{CS-U}^{*}||SK_{WD-U}\left|\right|R_{U}^{1}\left|\right|R_{4}\left|\right|T_{4})$ to verify the authenticity and integrity of the received message. If it matches, $U_{i}$ authenticates CS. After receiving the authentication confirmation message, $\left\{M_{9}\right\}$, $WD_{j}$ verifies $M_{9}^{*}\overset{?}{=}h(TID_{WD}\left|\right|R_{3}\left|\right|R_{4}||SK_{WD-U}^{*}\left|\right|T_{5})$ to verify the authenticity and integrity of the received message. If it is valid, $WD_{j}$ authenticates $U_{i}$. Thus, the AGPS-PUF successfully allows secure mutual authentication and integrity between $U_{i}$, $WD_{j}$, and CS.

#### 7.1.11. Anonymity and Untraceability

Assume that A intercepts the transmitted messages during the AKA phase. However, it is impossible for A to obtain $U_{i}$’s identity $ID_{U}$ and pseudo-identity $HID_{U}$ and $WD_{j}$’s identity $ID_{WD}$ and pseudo-identity $PID_{WD}$ without knowledge, such as random nonces, the PUF secret value, and secret credentials. Hence, the AGPS-PUF provides anonymity for $U_{i}$ and $WD_{j}$. Furthermore, A cannot track the legitimate $U_{i}$ since all messages are unique and dynamic using timestamps, random nonces, and temporary identities in each session. Moreover, the temporary identities, $TID_{U}$ and $TID_{WD}$ of $U_{i}$ and $WD_{j}$, are updated as $TID_{i}^{new}$ and $TID_{j}^{new}$ in each session. Hence, 3P-AGPS guarantees untraceability for $U_{i}$ and $WD_{j}$.

#### 7.1.12. Perfect Forward Secrecy (PFS)

The PFS security indicates that SK will not be exposed to A even if a long-term secret key is compromised. In the AGPS-PUF, if CS’s long-term secret key MK is compromised, A cannot compute the session keys, $SK_{CS-U}$ and $SK_{WD-U}$, because A does not have knowledge of the secret credentials $\left\{X_{UW},X_{i}\right\}$, the PUF secret value $\left\{R_{U}^{x},R_{WD}^{x}\right\}$, and real identities $\left\{ID_{U},ID_{WD}\right\}$. Consequently, the AGPS-PUF is resistant to PFS.

### 7.2. Formal Analysis through ROR Oracle Model

We utilize a formal proof, denoted as the ROR Oracle model, to prove the session key (SK) security. We define the queries required for the ROR Oracle model [10].

In the AGPS-PUF, there are three participants: the mobile user $\Gamma_{U}$, the wearable device $\Gamma_{WD}$, and the cloud server $\Gamma_{CS}$. Let $\Gamma_{U}^{t_{1}}$ be the instance $t_{1}$ of a participant *U*, $\Gamma_{WD}^{t_{2}}$ be the instance $t_{2}$ of a participant WD, and $\Gamma_{CS_{}}^{t_{3}}$ be the instance $t_{3}$ of a participant CS. In Table 2, we present the descriptions for each query, including “CorruptMD(·),Execute(·), Test(·), Send(·), and Reveal(·) for ROR Oracle model”.

**Theorem 1.**  *Let $Adv_{A}^{AGPS-PUF}$ be the advantage that $Adv_{A}^{AGPS-PUF}$ is able to break the SK security of the AGPS-PUF. Hence, we derive the following $Adv_{A}^{AGPS-PUF}\;\leq \;\frac{q_{h}^{2}}{\left|Hash\right|}+\frac{q_{P}^{2}}{\left|PUF\right|}+2\left\{C\cdot q_{send}^{s},\frac{q_{s}}{2^{l_{1}}},\frac{q_{s}}{2^{l_{2}}}\right\}$*

**Proof.**  The PUF(·) range space, h(·) query number, Send(·) query number, and Hash range space indicate $q_{P}$, $q_{h}$, $q_{send}$, and Hash. Furthermore, the Zipf credentials [39] indicate *C*, $l_{m}$, *s*, and $l_{n}$.

**Proof:** We present the five games $GM_{i}$ (i∈[0,4]). We indicate that $Adv_{A,GM_{i}}^{AGPS-PUF}$ is the probability of A to win $GM_{i}$. All games are described in detail as follows.

**Game:**$GM_{0}$: A executes a real attack in AGPS-PUF. Hence, A picks a random bit *c* at the beginning of $GM_{0}$. We obtain the following Equation (1) as

(1) $$Adv_{A}^{AGPS-PUF}=\left|2\cdot Adv_{A,GM_{0}}^{AGPS-PUF}-1\right|$$

**Game**$GM_{1}$: $GM_{1}$ indicates that A executes an “eavesdropping attack, in which the transmitted messages are intercepted between *U*, WD, and CS performing Execute(·) query”. In $GM_{1}$, A carries out “Test(·)/Reveal(·) queries” to compromise SK. The results of the Test(·)/Reveal(·) queries determine whether A obtains $SK_{CS-U}$ and $SK_{WD-U}$. To compromise SK, A requires the random nonces $\left\{R_{2},R_{3},R_{4}\right\}$, and PUF values. Therefore, A is not able to increase the winning probability of $GM_{1}$. We can derive Equation (2) as

(2) $$Adv_{A,GM_{1}}^{AGPS-PUF}=Adv_{A,GM_{0}}^{AGPS-PUF}$$

**Game**$GM_{2}$: This game indicates that A executes a “real attack” based on “Send(·) and Hash” queries. A transmits the modified messages to participants and acts as a legal user so that it is able to guess the outcomes of the “Send(·) query”. Moreover, A aims to find collisions for the hash oracle and attempts to copy messages that are expected to be authenticated by the entities. Because the random nonce, timestamp, temporary secret, and identity are configured using hash functions in each message, running “Send(·) and Hash queries” cannot cause a conflict. We can deduce that the probability of aborting the game is bounded by $\frac{q_{h}^{2}}{2|Hash|}$. It is worth noting that this may happen when processing Send() query; the game is aborted with a probability determined by the birthday paradox [40]. The probability of finding collisions in the hash oracle Hash, as per the square of the birthday paradox, is the probability, and the two games, $GM_{1}$ and $GM_{2}$, are indistinguishable, unless one of the above rules causes the game to abort. Thus, we can have Equation (3) as

(3) $$|Adv_{A,GM_{2}}^{AGPS-PUF}-Adv_{A,GM_{1}}^{AGPS-PUF}|\;\leq \;\frac{q_{h}^{2}}{2|Hash|}$$

**Game**$GM_{3}$: This game is executed in the analogy as presented in $GM_{2}$. By using the “analogous argument” described in $GM_{2}$, we can derive Equation (4) as

(4) $$|Adv_{A,GM_{3}}^{AGPS-PUF}-Adv_{A,GM_{2}}^{AGPS-PUF}|\;\leq \;\frac{q_{P}^{2}}{2|PUF|}$$

**Game**$GM_{4}$: In this game, A attempts to extract $\left\{A_{i},B_{i},D_{i},TID_{U}\right\}$ in the MD’s memory by using the “differential power analysis” with CourruptWD(·) and CourruptMD(·) queries. Note that $A_{i}=(X_{i}\left|\right|X_{UW})⊕h(HID_{U}⊕h(HPW_{i}\left|\right|R_{U}^{x}))$, $B_{i}=h(HPW_{i}||TID_{U}\left|\right|X_{i})$, $D_{i}=a_{i}⊕h(ID_{U}||PW_{i}\left|\right|R_{U}^{x})$, and $TID_{U}$. Moreover, A can obtain the secret credentials $\left\{E_{j},O_{j}\right\}$ in the WD’s memory using physical capture attacks. Note that, $E_{j}=(X_{UW}\left|\right|Z_{j}||PID_{WD})⊕h(ID_{WD}||TID_{WD}\left|\right|R_{WD}^{x}\left|\right|b_{j})$ and $O_{j}=b_{j}⊕h\left(R_{WD}^{x}⊕TID_{WD}⊕ID_{WD}\right)$. However, $GM_{3}$ is computationally infeasible for A to compromise the $PW_{i}$ of the legitimate $U_{i}$ over the Send(·) query without $a_{i}$ and $R_{U}^{x}$. Moreover, A should guess the parameters from the extracted data because A does not have knowledge of the “password”, “biometric”, and “PUF secret”. Moreover, it is computationally impossible to guess the “biometric”, “password”, and “PUF secret”. In conclusion, $GM_{3}$ and $GM_{4}$ are “indistinguishable”. We obtain Equation (5) as follows:

(5) $$|Adv_{A,GM_{4}}^{AGPS-PUF}-Adv_{A,GM_{3}}^{AGPS-PUF}|\;\leq \;\left\{C\cdot q_{send}^{s},\frac{q_{s}}{2^{l_{b}}}\right\}$$

Based on the execution of $GM_{0}-GM_{4}$, A attempts to guess the “bit *c* to win the games by performing Test(·) query”. We can obtain Equation (6) as follows:

(6) $$Adv_{A,GM_{4}}^{AGPS-PUF}=\frac{1}{2}$$

Based on the “Formulas (1), (2), and (6)”, we obtain Equation (7) as follows:

(7) $$\begin{array}{cc} \frac{1}{2}Adv_{A}^{AGPS-PUF} & =|Adv_{A,GM_{0}}^{AGPS-PUF}-\frac{1}{2}|\\ \; & =|Adv_{A,GM_{1}}^{AGPS-PUF}-\frac{1}{2}|\\ \; & =|Adv_{A,GM_{1}}^{AGPS-PUF}-Adv_{A,GM_{4}}^{AGPS-PUF}| \end{array}$$

Based on the “triangular inequality with the Formulas (3), (4), (5) and (7)”, we obtain Equation (8) as follows:

(8) $$\begin{array}{cc} \frac{1}{2}Adv_{A}^{AGPS-PUF} & =|Adv_{A,GM_{1}}^{AGPS-PUF}-Adv_{A,GM_{4}}^{AGPS-PUF}|\\ \; & \;\leq \;|Adv_{A,GM_{1}}^{AGPS-PUF}-Adv_{A,GM_{3}}^{AGPS-PUF}|\\ \; & \;+|Adv_{A,GM_{3}}^{AGPS-PUF}-Adv_{A,GM_{4}}^{AGPS-PUF}|\\ \; & \;\leq \;|Adv_{A,GM_{1}}^{AGPS-PUF}-Adv_{A,GM_{2}}^{AGPS-PUF}|\\ \; & \;+|Adv_{A,GM_{2}}^{AGPS-PUF}-Adv_{A,GM_{3}}^{AGPS-PUF}|\\ \; & \;+|Adv_{A,GM_{3}}^{AGPS-PUF}-Adv_{A,GM_{4}}^{AGPS-PUF}|\\ \; & \;\leq \;\frac{q_{h}^{2}}{2|Hash|}+\frac{q_{P}^{2}}{2|PUF|}+\left\{C\cdot q_{send}^{s},\frac{q_{s}}{2^{l_{1}}},\frac{q_{s}}{2^{l_{2}}}\right\}. \end{array}$$

Finally, by multiplying both sides of Equation (8) by a factor of 2, we can obtain the following: $Adv_{A}^{AGPS-PUF}\;\leq \;\frac{q_{h}^{2}}{\left|Hash\right|}+\frac{q_{P}^{2}}{\left|PUF\right|}+2\left\{C\cdot q_{send}^{s},\frac{q_{s}}{2^{l_{1}}},\frac{q_{s}}{2^{l_{2}}}\right\}$ □

### 7.3. Formal Analysis through AVISPA Simulation

This simulation proves the formal security robustness of the cryptographic protocol against MITM and replay attacks. We implement the security simulation and demonstrate the security result. We first need to implement the AGPS-PUF as a programming language HLPSL [41]. After that, this simulation starts analyzing the intermediate format (IF) over the four backends: “On-the-Fly Model Checker (OFMC)”, “Constraint Logic-based Attack Searcher (CL-AtSe)”, “SAT-based Model-Checker (SATMC)”, and “Tree Automata based on Automatic Approximations for the Analysis of Security Protocols (TA4SP)”. Since TA4SP and SATMC backends do not implement XOR operations, the simulation results of the AGPS-PUF under these backends become inconclusive; thus, the results based on TA4SP and SATMC backends have been ignored.

**Figure 6:**
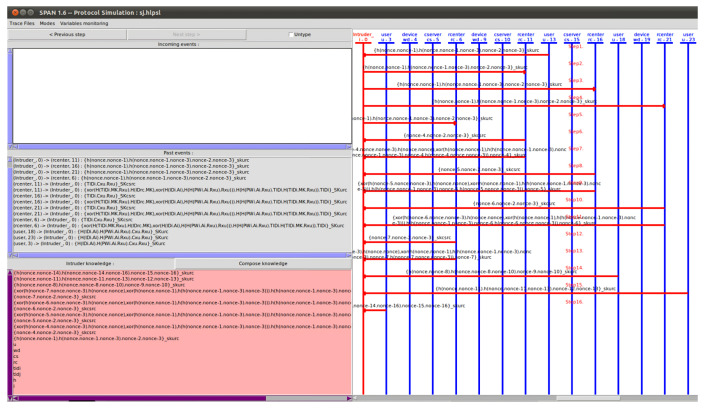
AVISPA simulation results based on SPAN.

We simulated the AGPS-PUF using the “Security Protocol ANimator (SPAN) [10]” for AVISPA. It is worth noting that AVISPA implements the DY model and that an intruder participates in the protocol execution with a concrete session. The specification roles of the WD, *U*, and CS are implemented using HLPSL, such as sessions, security goals, and environments. In Figure 6, the HLPSL specification of the protocol is converted into the IF by using the HLPSL2IF translator. After that, the IF is converted to the output format (OF) by feeding it to one of the four backends. The OF contains the following:

- SUMMARY: It refers to whether the tested security protocol is safe or unsafe, or whether the analysis is inconclusive.
- DETAILS: It explains why the analysis is inconclusive, why the tested security protocol is safe, or under what conditions the test applications or security protocols may be exploitable to the attack.
- PROTOCOL: It refers to the HLPSL specification of the target security protocol in the IF.
- GOAL: It demonstrates the goal of the analysis, which is performed by AVISPA using HLPSL specifications.
- BACKEND: It is the name of the backend that is utilized for the analysis of SATMC, CL-AtSe, OFMC, or TA4SP.
- STATISTICS: It includes the trace of any potential vulnerability in the target security protocol, along with several useful statistics and related comments.

**Figure 7:**
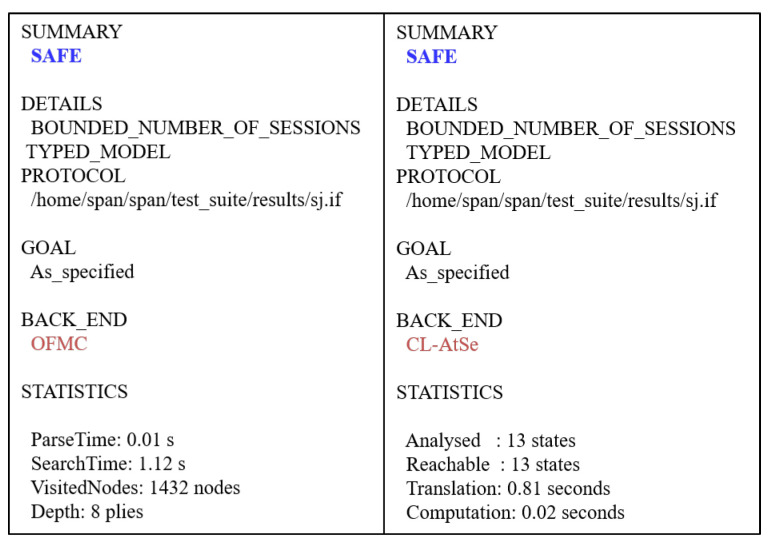
AVISPA results based on OFMC and CL-AtSe.

In the simulation based on AVISPA backends, two verifications were performed: (1) checking for replay attacks and (2) DY model-based MITM attacks. When checking for replay attacks on the AGPS-PUF, both OFMC and CL-AtSe check if the legitimate participants can execute the specified protocols by performing a search for a passive intruder. Moreover, both OFMC and CL-AtSe backends are used to check whether any MITM attacks are possible by an intruder in the DY model. The SPAN simulation results demonstrate the security attacks and intruder simulations over a web-based GUI (graphical user interface). Moreover, the implementation results obtained using the CL-AtSe and OFMC backends are presented in Figure 7. According to the simulation results under the OFMC and CL-AtSe in Figure 7, the SAFE output shows that the AGPS-PUF is safe based on the specified security goals. Consequently, we demonstrate that the AGPS-PUF is protected from replay and MITM attacks.

## 8. Testbed Experiments Using MIRACL

We present the testbed experiments to estimate the execution times required for essential cryptographic operations utilized in the AGPS-PUF and existing related schemes. We used the well-known “MIRACL crypto SDK [42]”, which is a C/C++-based programming software library.

We used the two platforms to estimate the execution times required for cryptographic operations. $T_{sed}$, $T_{ecpm}$, $T_{me}$, and $T_{h}$ evaluate the execution times required for “a AES encryption and decryption”, “an ECC scalar point multiplication”, “a modular exponentiation”, and “a SHA-256 hash function”.

- **Platform 1:** This platform is used to calculate the execution times for the MD and WD settings on MIRACL, as follows: “Model: Raspberry PI 4B, with “OS: Ubuntu 20.04.2 LTS”, “Processor: 1.5 GHz Quad-core”, “CPU: 64-bit”. Each operation was run 1000 times on the same setup and we observed the average, maximum, and minimum times. The results of this platform are tabulated in Table 3.

- **Platform 2:** This platform was used to calculate the execution time for the CS server setting as follows: “OS: Ubuntu 18.04.4 LTS, Processor: Intel Core i5-10400 @2.9 GHz, Six-core, CPU: 64-bits”. All primitives were run 1000 times on the same setup and we observed the average, maximum, and minimum times. The results of this platform are tabulated in Table 4.

## 9. Performance Comparison

This section presents the “performance comparison analysis” of the AGPS-PUF and existing related schemes for wearable computing [8,21,23,25,26,28].

### 9.1. Computation Costs

We discuss the comparative computation costs of the AGPS-PUF with the existing related schemes [8,21,23,25,26,28] during the AKA phase. We used the“ testbed experimental results for the Raspberry PI 4 and server setting in Section 8”. With the information presented in Table 3, we utilized the analysis results of the average time for each operation under $U_{i}$ and $WD_{j}$.

**Figure 8:**
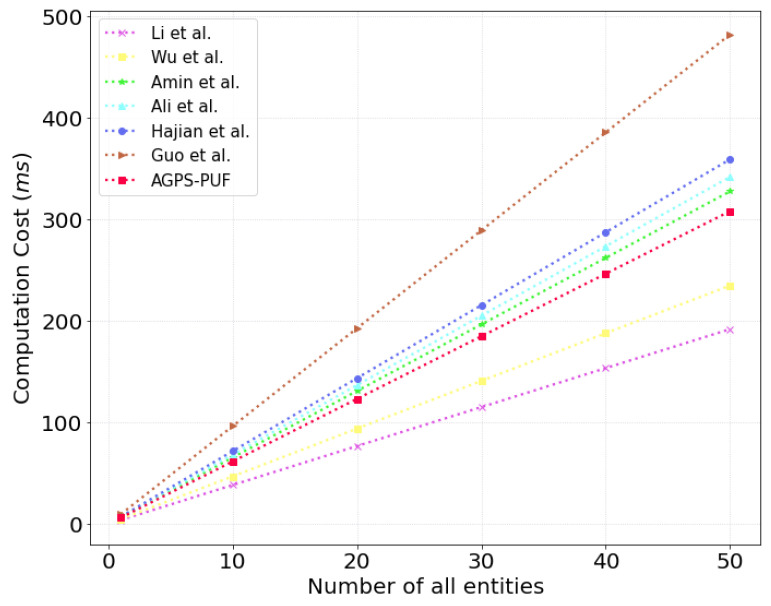
Computational cost comparison of all entities.

We calculated the execution times for the MD and WD settings on MIRACL as follows: “Model: Raspberry PI 4B, with “OS: Ubuntu 20.04.2 LTS”, “Processor: 1.5 GHz Quad-core”, “CPU: 64-bit”. As seen in Table 3, we present “$T_{ecpm}\approx 2.848$ ms, $T_{h}\approx 0.309$ ms, $T_{me}\approx 0.228$ ms and $T_{sed}\approx 0.012$ ms”. Moreover, we calculated the execution times for the CS server setting as follows: “OS: Ubuntu 18.04.4 LTS, Processor: Intel Core i5-10400 @2.9 GHz, Six-core, CPU: 64-bits”. As seen in Table 4, we utilized the analysis results for the average time of each operation under CS. In scenario 2, we present “$T_{ecpm}\approx 0.522$ ms, $T_{h}\approx 0.055$ ms, $T_{me}\approx 0.040$ ms and $T_{sed}\approx 0.001$ ms”. We prove the performance results for the comparative computational costs in Table 5 and Figure 8. Consequently, the AGPS-PUF offers the necessary security requirements and features while maintaining similar costs compared to previous schemes [8,25,26,28]. Hence, the AGPS-PUF is suitable for practical wearable computing environments.

### 9.2. Communication Costs

**Figure 9:**
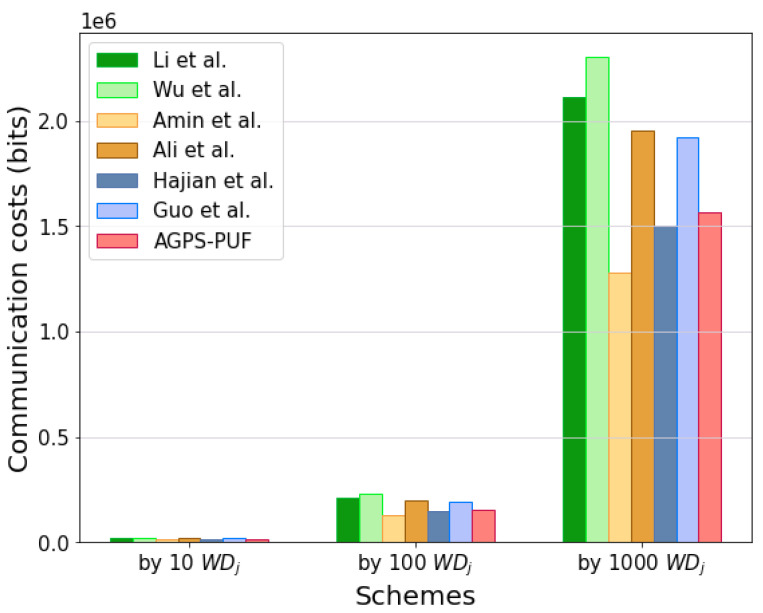
Communication cost comparison.

We discuss the comparative communication costs of the AGPS-PUF and existing related schemes [8,21,23,25,26,28]. Referring to [8], we assume that the bits for the timestamp, the PUF challenge, identity, random nonce, symmetric encryption/decryption, and hash digest are 32, 64, 128, 128, 128, and 256 bits, respectively. During the AKA phase of the AGPS-PUF, the exchanged messages $\left\{M_{1},T_{1}\right\}$, $\left\{M_{2},M_{3},TID_{WD},C_{WD}^{1},T_{2}\right\}$, $\left\{M_{2},M_{3},M_{4},M_{5},TID_{U},TID_{WD},C_{WD}^{1},C_{U}^{1},T_{2},T_{3}\right\}$, $\left\{M_{6},M_{7},T_{4}\right\}$, and $\left\{M_{8},M_{9},TID_{j}^{*},T_{4},T_{5}\right\}$ require “(256 + 32 = 288 bits), (256 + 256 + 128 + 64 + 32 = 736 bits), (256 + 256 + 128 + 256 + 128 + 128 + 64 + 64 + 32 + 32 = 1344), (128 + 256 + 32 = 416 bits), and (256 + 256 + 256 + 32 + 32 = 832 bits)”. Consequently, the AGPS-PUF has similar costs compared with previous schemes, as presented in Table 6 and Figure 9, since transmitting fewer bits minimizes the network latency and number of collisions.

### 9.3. Security Functionality Comparison

This section compares the “security functionalities” of the AGPS-PUF with the existing related schemes for wearable computing [8,21,23,25,26,28]. In Table 7, we show that some existing schemes for wearable computing are not fully protected and may be fragile to different potential security attacks. Thus, the security protocols must be designed in such a way that they must be robust against lethal security attacks. In contrast, the AGPS-PUF is resilient to lethal security attacks, and guarantees the necessary security requirements and functionalities, including “mutual authentication, PFS, anonymity, and untraceability”. Thus, the AGPS-PUF provides more security functionalities when compared to the existing related schemes for wearable computing [8,21,23,25,26,28].

## 10. Conclusions

We prove that Guo et al.’s scheme is not protected against session key disclosure, MITM, and impersonation attacks, and it does not offer security requirements and features such as mutual authentication and untraceability. Hence, we designed an efficient and robust authentication and group–proof scheme using the PUF for wearable computing to address the security issues of Guo et al.’s scheme. We demonstrate the session key security of the AGPS-PUF by performing formal security under the ROR Oracle model analysis and show that the AGPS-PUF is resistant to replay and MITM attacks by using the AVISPA simulation analysis. Furthermore, we present the testbed experiments of the AGPS-PUF using MIRACL crypto SDK based on Raspberry PI 4. We demonstrate the performance comparison of the AGPS-PUF and the existing related schemes for wearable computing with respect to computation costs, communication costs, and security features. Thus, the AGPS-PUF ensured a higher security level than the existing related scheme in wearable computing environments and provided similar computational and communication costs to the existing related schemes for wearable computing. Thus, the AGPS-PUF is suitable for practical wearable computing environments, as it offers more effective efficiency and superior security compared to existing related schemes for wearable computing.

## Figures and Tables

**Table 1:** Notations.

Symbol	Meaning
$U_{i}$	*i*th user
$WD_{j}$	*j*th wearable device
CS	Cloud server
RC	Registration center
$ID_{U},ID_{WD},ID_{CS},ID_{RC}$	Real identity of $U_{i}$, $WD_{j}$, CS, and RC
$PW_{i}$	Password of $U_{i}$
$C_{U}^{x},R_{U}^{x}$	Challenge/response of $U_{i}$
$C_{WD}^{x},R_{WD}^{x}$	Challenge/response of $WD_{j}$
$R_{i},RN_{i},n_{i}$,	Random nonce
$TID_{U},TID_{WD}$,	Temporary identity of $U_{i}$ and $WD_{j}$
$\Delta T_{i}$	Maximum transmission delay
$T_{i}$ and $TS_{i}$	Timestamp
MK	A master private key of CS
$SK_{CS-U}$	A session key for $U_{i}$ and CS
$SK_{WD-U}$	A session key for $U_{i}$ and $WD_{j}$
$Enc_{K}\left(\cdot \right)/Dec_{K}\left(\cdot \right)$	Encryption/decryption
h(·)	Hash function
⊕	XOR function
||	Concatenation

**Table 2:** Query and purpose.

Query	Purpose
Send $\left(\Gamma^{t},Msg\right)$	Based on this query, A can transmit the message Msg to the $\Gamma^{t}$, and obtain the response message accordingly.
CorruptMD $\left(\Gamma_{U}^{t_{1}}\right)$	This query indicates as the mobile device stolen attacks, where A can extract the secret credentials stored in MD.
CorruptWD $\left(\Gamma_{WD}^{t_{3}}\right)$	This query indicates as the physical capture attacks where A can obtain the secret parameters stored in WD.
$Execute(\Gamma_{U}^{t_{1}},$$\Gamma_{WD}^{t_{2}},$$\Gamma_{CS}^{t_{3}},$)	Based on Execute(·), A performs the passive/active attacks by eavesdropping the exchanged messages between each entity over a insecure channel.
$Reveal(\Gamma^{t})$	Based on this query, A reveals a SK generated between $\Gamma_{U}^{t_{1}}$ and $\Gamma_{WD}^{t_{2}}$ using Reveal(·) query.
$Test(\Gamma^{t})$	An unbiased coin *c* is tossed prior to game start. If A gets c=1 under the Test(·), it indicates a SK between $\Gamma_{U}^{t_{1}}$ and $\Gamma_{WD}^{t_{2}}$ is fresh. If A gets the c=0, it indicates SK is not fresh; otherwise, A gets a null value (⊥).

**Table 3:** Execution times (in milliseconds) based on the MIRACL library, obtained using a Raspberry Pi 4.

Operation	Min. Time (ms)	Max. Time (ms)	Average Time (ms)
$T_{ecpm}$	2.766	2.920	2.848
$T_{h}$	0.274	0.643	0.309
$T_{me}$	0.178	0.493	0.228
$T_{sed}$	0.011	0.021	0.012

**Table 4:** Execution times (in milliseconds) based on the MIRACL library for a server.

Operation	Min. Time (ms)	Max. Time (ms)	Average Time (ms)
$T_{ecpm}$	0.472	2.737	0.522
$T_{h}$	0.024	0.149	0.055
$T_{me}$	0.022	0.082	0.040
$T_{sed}$	0.001	0.002	0.001

**Table 5:** Comparison between computational costs.

Scheme	User	Gateway/Server	Wearable Device	Total Computation Cost
Li et al. [21]	$6T_{h}+2T_{sed}$	$7T_{h}+6T_{sed}$	$5T_{h}+2T_{sed}$	$18T_{h}+10T_{sed}$
Wu et al. [23]	$10T_{h}+2T_{sed}$	$6T_{h}+5T_{sed}$	$4T_{h}+T_{sed}$	$20T_{h}+8T_{sed}$
Amin et al. [25]	$12T_{h}$	$18T_{h}$	$6T_{h}$	$36T_{h}$
Ali et al. [26]	$12T_{h}+2T_{sed}$	$16T_{h}+3T_{sed}$	$7T_{h}+5_{sed}$	$35T_{h}+10T_{sed}$
Hajian et al. [28]	$13T_{h}$	$7T_{h}$	$9T_{h}$	$29T_{h}$
Guo et al. [8]	$21T_{h}$	$18T_{h}$	$7T_{h}$	$46T_{h}$
AGPS-PUF	$10T_{h}+3T_{sed}$	$10T_{h}+2T_{sed}$	$8T_{h}+T_{sed}$	$28T_{h}+6T_{sed}$

**Table 6:** Comparison between communication costs.

Scheme	Communication Cost for ${\mathrm{WD}}_{j}$	Total Cost	Number of Messages
[21]	2112 bits	4352 bits	4 messages
[23]	2304 bits	2816 bits	3 messages
[25]	1280 bits	4096 bits	5 messages
[26]	1952 bits	4128 bits	4 messages
[28]	1504 bits	3552 bits	5 messages
[8]	1920 bits	5088 bits	5 messages
AGPS-PUF	1568 bits	3616 bits	5 messages

**Table 7:** Comparative study on security features.

Security Features	[21]	[23]	[25]	[26]	[28]	[8]	Our
$SF_{1}$	o	o	o	o	x	x	o
$SF_{2}$	o	x	x	o	o	o	o
$SF_{3}$	o	x	x	o	x	x	o
$SF_{4}$	x	NA	NA	NA	o	x	o
$SF_{5}$	NA	x	x	NA	x	o	o
$SF_{6}$	o	o	o	o	o	o	o
$SF_{7}$	o	o	x	o	x	x	o
$SF_{8}$	o	o	o	o	o	o	o
$SF_{9}$	x	x	x	o	o	o	o
$SF_{10}$	o	o	o	o	x	x	o
$SF_{11}$	x	x	x	o	o	o	o
$SF_{12}$	o	o	o	o	o	o	o
$SF_{13}$	o	x	o	o	o	x	o
$SF_{14}$	x	o	o	o	o	x	o
$SF_{15}$	NA	NA	NA	NA	NA	o	o

o: “protection of security features”; x: “non-protection of security features”; NA: “not applicable”; $SF_{1}$: “MITM attack”; $SF_{2}$: “offline password-guessing attack”; $SF_{3}$: “impersonation attack”; $SF_{4}$: “sensor physical capture attack”; $SF_{5}$: “mobile device stolen attack”; $SF_{6}$: “stolen verifier attack”; $SF_{7}$: “session key disclosure attack”; $SF_{8}$: “replay attack”; $SF_{9}$: “privileged insider attack”; $SF_{10}$: “mutual authentication”; $SF_{11}$: “user anonymity”; $SF_{12}$: “PFS”; $SF_{13}$: “untraceability”; $SF_{14}$: “formal (simulation) analysis”; $SF_{15}$: “group proof”.

## Data Availability

Not applicable.
